# Pharmacology of nivegacetor (RG6289), a potent and selective gamma secretase modulator in clinical development for the treatment of Alzheimer’s disease

**DOI:** 10.3389/fphar.2026.1783414

**Published:** 2026-05-28

**Authors:** Lothar Lindemann, Julie Lambotte, Judith Rothe, Jürg Messer, Catherine Diener, Solen Pichereau, Carina Cantrill, Thomas Mueggler, Michael Honer, Jennifer Beck, Thomas Steinbrecher, Rosanna Tortelli, Irene Gerlach, Hasane Ratni, Rosa Maria Rodriguez Sarmiento, Karlheinz Baumann

**Affiliations:** 1 Roche Pharma Research & Early Development, Neuroscience and Rare Diseases Translational Area, Discovery Neuroscience, F. Hoffmann-La Roche AG, Roche Innovation Center Basel, Basel, Switzerland; 2 Roche Pharma Research & Early Development, Pharmaceutical Sciences, F. Hoffmann-La Roche AG, Roche Innovation Center Basel, Basel, Switzerland; 3 Roche Pharma Research & Early Development, Neuroscience and Rare Diseases Translational Area, Biomarkers, F. Hoffmann-La Roche AG, Roche Innovation Center Basel, Basel, Switzerland; 4 Roche Pharma Research & Early Development, Therapeutic Modalities, Small Molecules Research, Computer Aided Drug Design, F. Hoffmann-La Roche AG, Roche Innovation Center Basel, Basel, Switzerland; 5 Roche Pharma Research & Early Development, Neuroscience and Rare Diseases Translational Area, Early Clinical Development, F. Hoffmann-La Roche AG, Roche Innovation Center Basel, Basel, Switzerland; 6 Roche Pharma Research & Early Development, Therapeutic Modalities, Small Molecules Research, Medicinal Chemistry, F. Hoffmann-La Roche AG, Roche Innovation Center Basel, Basel, Switzerland

**Keywords:** Alzheimer’s disease, amyloid beta, disease-modifying therapy, gamma secrease modulator, gamma secretase, GSM, nivegacetor, RG6289

## Abstract

**Background:**

Alzheimer’s Disease (AD) is a prevalent neurodegenerative disorder which involves a complex pathobiology driven by amyloid-beta (Aβ) and tau pathologies, among other factors. Aβ peptides are generated *via* β-secretase (BACE1) and γ-secretase cleavage of amyloid precursor protein (APP). While long isoforms like Aβ42 are neurotoxic and aggregation-prone, shorter isoforms (Aβ38, Aβ37) are non-amyloidogenic. γ-secretase modulators (GSMs) shift production from longer to shorter peptides which is expected to slow down or halt (prevent) amyloid accumulation and its downstream effects.

**Methods:**

The novel GSM nivegacetor was evaluated *in vitro* using cell lines overexpressing human wild-type APP, or human APP with the Swedish mutation K670N/M671L (APPSwe). The *in vitro* selectivity of nivegacetor was tested on Notch-1, a representative gamma secretase substrate other than APP. Additionally, nivegacetor was profiled for its selectivity on a range of pharmacological targets. *In vivo* studies tested a dose-response and a time course of nivegacetor on soluble Aβ levels in brain tissue of APPSwe transgenic mice. Furthermore, the impact of two ADAD mutations, PSEN1 E280A (Columbian) and PSEN2 N141I (Volga German), on nivegacetor’s potency was tested. Moreover, nivegacetor was tested for possible effects on [^3^H]florbetaben binding to Aβ plaque pathology in human AD brain tissue sections.

**Results:**

Nivegacetor lowered the production of Aβ42 and Aβ40 and concomitantly increased levels of Aβ37 and Aβ38 *in vitro* and *in vivo* in mice. Nivegacetor did not inhibit Notch-1 and showed a favorable selectivity profile on a broad range of targets. When tested on two ADAD mutations, nivegacetor was equipotent on the PSEN1 E280A mutation and significantly less potent on the PSEN2 N141I mutation compared to wild-type gamma secretase. Nivegacetor did not interfere with the detection of amyloid plaques by [^3^H]florbetaben in human AD brain tissue, which is an important prerequisite for the use of florbetaben as a PET tracer in clinical trials.

**Conclusion:**

Nivegacetor is a potent, orally bioavailable GSM with favorable properties and is currently under investigation as a clinical candidate in a Phase 2A clinical trial in individuals with prodromal and early sporadic AD, and in a Phase 2 clinical trial in individuals carrying the PSEN1 E280A ADAD mutation.

## 1 Introduction

Alzheimer’s Disease (AD) is the leading cause of dementia and a major public health burden (Chen et al., 2024; Xiaopeng et al., 2025). It is a complex disease that starts with clinically silent changes, often decades before biomarkers and the clinical presentation of individuals prompt a diagnosis. The underlying pathobiology of AD involves the well-known amyloid beta (Aβ) and tau pathologies, lipoproteins, and inflammatory processes that eventually lead to neuronal dysfunction, the loss of synapses and neurons, and to the clinical manifestations of the disease (De Strooper and Karran, 2016; Knopman et al., 2021; Scheltens et al., 2021).

The essential role of Aβ in the AD pathobiology is apparent from the fact that there is no AD without Aβ pathology, even though individuals with high amyloid load can remain asymptomatic over long periods of time. Moreover, Autosomal Dominant Alzheimer’s Disease (ADAD) mutations, which cause an increased rate of Aβ production and a shift towards a higher fraction of long Aβ fragments such as Aβ42 (Szaruga et al., 2017; Trambauer et al., 2020), are linked to forms of AD with a rapid disease progression and usually with an early onset (Hoogmartens et al., 2021). On the contrary, genetic mutations leading to lower Aβ levels such as the ‘Icelandic’ mutation APP A673T have a protective effect against AD (Jonsson et al., 2012; Wittrahm et al., 2023).

**FIGURE 1:**
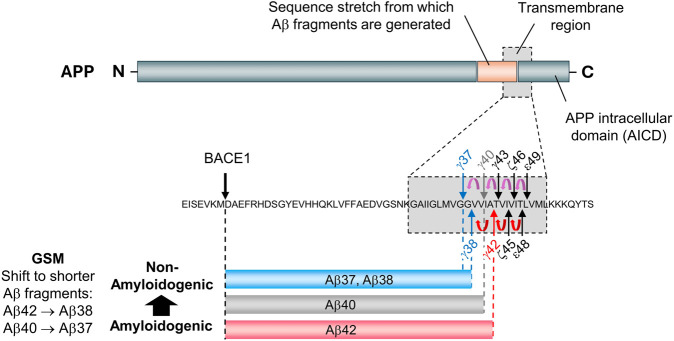
Overview of Aβ fragment production following APP processing by BACE1 and gamma secretase, and effects of gamma secretase modulators on Aβ fragment lengths. APP undergoes an initial processing by BACE1 and gamma secretase, producing long precursor peptides of 49 and 48 amino acids length which remain bound to the gamma secretase complex. These precursor peptides undergo exoproteolytic trimming by gamma secretase until they are released as a blend of Aβ fragments with different lengths. Under non-disease conditions the most common fragments are: Aβ40 (∼80%), Aβ42 (∼10%), Aβ38 (∼10%), and Aβ37, and other fragment lengths at very low abundance or under pathological conditions. In the presence of a gamma secretase modulator (GSM) the Aβ peptides remain bound to the gamma secretase enzyme until the trimming has produced shorter fragments sizes, i.e., Aβ38 instead of Aβ42 and Aβ37 instead of Aβ40 is released. Diagram with modifications based on Karran et al. (2011), with information from Steiner et al. (2018).

The significance of Aβ as a drug target is underscored by the success of Aβ immunotherapies which slow disease progression by lowering the amyloid load in AD patients (Sims et al., 2023; van Dyck et al., 2023). While these therapies effectively clear existing amyloid plaques, they do not alter the *de novo* production of Aβ. Beta-secretase 1 (BACE1) and gamma secretase are the critical proteases involved in the processing of amyloid precursor protein (APP) and the production of Aβ fragments (Figure 1). Furthermore, the majority of the above-mentioned ADAD mutations are located in genes encoding subunits of the gamma secretase complex (PSEN1 and 2) and, to a lesser extent, APP (Karch and Goate, 2015; Steiner et al., 2018), highlighting the relevance of gamma secretase as a drug target for AD.

Gamma secretase is a multimeric protease complex composed of four subunits including presenilin (PSEN; as two possible isoforms, PSEN1 or PSEN2), nicastrin, anterior pharynx defective 1 (APH-1), and presenilin enhancer 2 (PEN-2); the catalytically active site is located in the PSEN subunit (De Strooper et al., 2012). Of note, gamma secretase is involved in the proteolytic processing of many substrates including APP, Notch-1, Lrp6, IGF1-R, or Trem2 which play important roles in a range of different biological processes (Guner and Lichtenthaler, 2020; Hou et al., 2023; Breimann et al., 2025). Similarly, BACE1 is involved in the processing of several different targets including APP, voltage-gated sodium channels, and cell adhesion molecules (Hemming et al., 2009). Thus, it is not surprising that inhibitors of BACE1 and gamma secretase, while effective in lowering Aβ, turned out to be not viable as approaches for treating AD (Bazzari and Bazzari, 2022; Wolfe, 2024).

With respect to APP processing, typically a blend of different Aβ fragment lengths is produced, most commonly Aβ40 and Aβ42, as well as Aβ37 and Aβ38 and also other fragment lengths albeit at lower abundance or only under pathological conditions (Steiner et al., 2018; Hur, 2022). In terms of abundance under non-pathological conditions, Aβ40 is by far the most abundant isoform, followed by Aβ42 representing about 10% of the total Aβ and smaller amounts of Aβ37 and Aβ38 (Steiner et al., 2018). The Aβ peptides differ in their biological effects. Aβ42 (and longer fragments) have toxic effects and are prone to aggregate and drive the production of Aβ plaques (Selkoe and Hardy, 2016). The shorter fragments Aβ37 and Aβ38 do not aggregate and can inhibit the formation of Aβ aggregates by acting as β-sheet brakers (Braun et al., 2022). Higher concentrations of Aβ38 in CSF have been associated with a slower cognitive decline and a lower risk of developing AD dementia in two independent observational clinical trials (Cullen et al., 2022). Furthermore, lower concentrations of shorter Aβ peptides in CSF correlate with a younger age of symptoms onset (Fernandez et al., 2025), with higher scores on the Clinical Dementia Rating Scale-Sum of Boxes, and lower scores on the Mini Mental State Examination (Schultz et al., 2024) in ADAD. Gamma secretase modulators (GSMs) alter the processivity of the gamma secretase complex so that fewer long Aβ fragments (Aβ42 and Aβ40) and more shorter Aβ fragments (Aβ37 and Aβ38) are produced (De Strooper and Karran, 2024). Importantly, GSMs do not alter the total amount of Aβ fragments being produced, and they do not inhibit the enzyme activity and thus do not affect the processing of substrates other than APP. Therefore, GSMs are considered to be a promising therapeutic strategy for AD (Nordvall et al., 2023; De Strooper and Karran, 2024).

## 2 Materials and methods

### 2.1 Study drugs and chemicals

**FIGURE 2:**
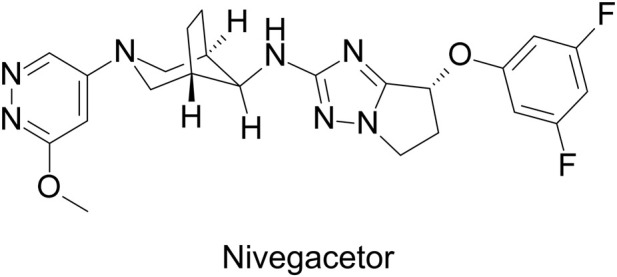
Chemical structure of nivegacetor.

Nivegacetor (C23H25F2N7O2, MW 469.497; RO7269162, RG6289; Figure 2) (Ratni and Frei, 2020) was synthesized at F. Hoffmann-La Roche AG and stored as dry powder at room temperature protected from light. Compounds were dissolved in dimethylsulfoxide (DMSO) at a stock concentration of 10 mM and stored at −20 °C. The gamma secretase inhibitor (GSI) RO4929097 (2,2-dimethyl-N-((S)-6-oxo6,7-dihydro-5H-dibenzo[b, d]azepin-7-yl)-N-(2,2,3,3,3-pentafluoro-propyl)-malonamide; C22H20F5N3O3, MW 469.407) (Luistro et al., 2009) was synthesized at F. Hoffmann-La Roche AG. The GSI DBZ (dibenzazepine; N-[(1S)-2-[[(7S)-6,7-Dihydro-5-methyl-6-oxo-5H-dibenz[b,d]azepin-7-yl]amino]-1-methyl-2-oxoethyl]-3,5-difluorobenzeneacetamide) was purchased at Tocris (Bristol, UK). The GSI MRK-560 (N-[cis-4-[(4-chlorophenyl)sulfonyl]-4-(2,5-difluorophenyl)cyclohexyl]-1,1,1-trifluoromethanesulfonamide; C19H17ClF5NO4S2, MW 517.921) (Churcher et al., 2006) used as reference compound for *in vivo* dose-response studies was purchased from Seleckchem (Houston, United States). Florbetapir was purchased at MedChemExpress (Monmouth Junction, United States). Compounds were dissolved in DMSO at a stock concentration of 10 mM and stored at −20 °C.

[^3^H]Florbetaben was synthesized at RC TRITEC AG (Switzerland), with a molar activity of 83.2 Ci/mmol and a radiochemical purity of 98%. The tracer was provided as an ethanolic stock solution and stored at −20 °C protected from light. The radiochemical purity confirmed at Roche at delivery was 70.3%; the radiochemical purity at the time of the experiment was 65.5% and, after repurification, was 66.3%.

All other chemical reagents were purchased from vendors such as Sigma, Merck, Tocris, and Invitrogen at the highest available quality.

### 2.2 Cell lines

All cell lines used in this study are based on H4 cells (Arnstein et al., 1974), HEK293 cells (Graham et al., 1977), and N2A cells (Olmsted et al., 1970) which were sourced from ATCC (cell lines HTB-148, CRL-1573, and CCL-131, respectively; American Type Culture Collection Manassas, Virginia, United States). H4 is a human neuroglioma cell line. HEK293 cells are a widely used human embryonic kidney cell line with epithelial cell characteristics. N2A cells are a murine neuroblastoma cell line. The 3 cell lines express endogenous, functional human or murine gamma secretase complex.

#### 2.2.1 HEK293 and N2A cell lines

For the production of clonal stably transfected cell lines, wild-type (WT) cells were transfected using lipofectamine 2000 (Invitrogen, Carlsbad, United States) essentially as described by the manufacturer. In brief, cells were plated the day before the transfection at a density of approximately 30%–50% and then treated with the lipofectamine/plasmid mixture as suggested by the manufacturer. About 24 h after the transfection, cells were detached and seeded into 96-well plates by limited dilution with approximately one single cell per well in cell culture medium containing the antibiotic used for selection. Surviving cell clones were expanded, tested for expression of the transgene, and a well-performing cell clone was banked for future use.

To produce cell lines expressing human APP, a cDNA encoding human APP-695 (A33292.1) was cloned into a pCDNA3.1 Hygromycin expression plasmid. A clonal stably transfected HEK293 cell line was produced with this plasmid using the lipofectamine transfection procedure as described above; this cell line was named HEK293 APPWT.

The cDNA encoding APP-695 (A33292.1) was subjected to site-directed mutagenesis by PCR, exchanging bases GA at position +1785 and +1786 to TC, which causes two changes in the amino acid sequence K670N and M671L (Lo et al., 1994); this cDNA was cloned into a pCDNA3.1 Hygromycin expression plasmid. This plasmid was used to produce clonal stably transfected N2A cell lines called N2A APPSwe.

For the Notch-1 selectivity assay, a gene reporter cell line was used (BPS Bioscience, Inc., San Diego, United States). This cell line stably expresses Notch1ΔE (NOTCH1 with a deletion of the entire extracellular domain) and the firefly luciferase gene under the control of Notch-response elements (CSL responsive elements: CBF1/RBPJκ/Suppressor of Hairless/Lag-1) and coactivator Mastermind (Lu and Lux, 1996). This cell line was named HEK-Notch-CSL-Luc.

#### 2.2.2 H4 cell lines

H4 cells stably expressing APP695 with the Swedish mutation were produced essentially following the procedure described for HEK293 cells using lipofectamine 2000 as transfection reagent. A clonal H4 cell line stably expressing APP695 Swedish was called H4 APPSwe31-10 (abbreviated H4 APPSwe).

For some of the cell lines used to characterize the effect of ADAD mutations a clonal double-knock-out cell line with targeted disruptions of PSEN1 and PSEN2 was created using Crispr/Cas technology, using the H4 APPSwe cell line described above as the basis; this cell line was named #B3 H4APPSwe31-10 PSEN1/PSEN2 KO. The Crispr/Cas KO cell line was produced by Synthego (San Diego, United States). The disruption of both PSEN1 and PSEN2 genes was confirmed by DNA sequencing (carried out at Synthego), which was corroborated by confirming the lack of Aβ sequestration into the cell culture medium (Supplementary Figure S1).

For the characterization of ADAD mutations, plasmids encoding WT PSEN1 (NP_000012) and PSEN1 E280A (‘Columbian’ mutation), as well as WT human PSEN2 (NP_000438) and PSEN2 N141I (‘Volga German’ mutation) were produced by Trenzyme (Konstanz, Germany). cDNAs encoding the WT or mutated forms of the proteins with optimized codon usage and KOZAK sequence were prepared by gene synthesis and cloned into the pIRES-NEO2 plasmid (Takara Bio, London, UK). The pIRES-NEO2 plasmid encodes a bicistronic cDNA comprising the gene of interest linked to the cDNA encoding neomycin resistance by an internal ribosomal entry site (IRES) which reduces the frequency of false positive cell clones that are resistant to neomycin but do not express the gene of interest.

For stable transfections of the H4 APPSwe and the #B3 H4APPSwe31-10 PSEN1/PSEN2 KO cell lines with plasmids encoding WT and mutant forms of presenilins the nucleofection technique was applied. In brief, the plasmids were transfected into H4 APPSwe or into #B3 H4APPSwe31-10 PSEN1/PSEN2 KO cells using the Nucleofector (Lonza Biosciences, Switzerland) essentially following the manufacturer’s instructions. For each transfection, 1 Mio cells were transferred to a 1.5 mL Eppendorf tube and centrifuged at 90 *g* at room temperature (RT) for 10 min. The supernatant was aspirated, and the cell pellet was resuspended by pipetting up and down in 100 µL Nucleofector solution from the SF Cell Line 4D-Nucleofector X kit. A total of 2 µg plasmid DNA was added, and the cell suspension was mixed by pipetting up and down once. The cell suspension was transferred into the Nucleofector cuvette which was inserted into the 4D-Nucleofactor TM X Unit and the program CM-138 with solution SF was run. The cells were subjected to a limited dilution in cell culture medium and plated in 96-well plates such that on average a single cell ended up per well. Cells were cultured in selection medium containing 300 μg/mL G418 (see Table 1 for cell culture medium composition) in humidified cell culture incubator at 37 °C with 5% CO_2_. Cell clones were expanded from 96-well format into increasingly larger cell culture vessels and characterized for the expression of Aβ peptides based on which the successful clonal cell lines were selected.

**TABLE 1:** Cell line names, transgene expression/genetic modifications, and cell culture media for cell lines used in this study.

Name (shorthand, where applicable)	Based on	Stable transgene expression/genetic modification	Cell culture medium
H4 APPSwe31-10 (H4 APPSwe)	H4	• APP695 Swedish	• IMDM + 10% FBS • 200 μg/mL hygromycin
#B3 H4 APPSwPS1PS2KO (H4 KO APPSwe)	H4	• H4 APPSwe cells with a homozygous targeted disruption of PSEN1 and PSEN2 genes	• IMDM + 10% FBS • 200 μg/mL hygromycin
N2A APPSwe	N2A	• APP695 Swedish	• IMDM + 10% FBS • 600 μg/mL G418
HEK293 APPWT	HEK293	• APP695	• IMDM + 10% FBS • 100 μg/mL G418
H4 APPswe31-10 + PS1(WT) #15 (H4 APPSwe + WT PS1)	H4	• APP695 Swedish • WT PSEN1	• IMDM + 10% FBS • 200 μg/mL hygromycin • 300 μg/mL G418
H4 APPswe31-10 + PS1(E280A) #25 (H4 APPSwe + PS1 E280A)	H4	• APP695 Swedish • PSEN1 E280A	
H4 APPSwe31-10 + PS2(WT) #3 (H4 APPSwe + WT PS2)	H4	• APP695 Swedish • WT PSEN2	
H4 APPSwe31-10 + PS2(N141l) #4 (H4 APPSwe + PS2 N141I)	H4	• APP695 Swedish • PSEN2 N141I	
#B3 H4 APPSwPS1PS2KO + PS1(WT) #17 (H4 KO APPSwe + WT PS1)	H4	• PSEN1 + PSEN2 Crispr/Cas KO • APP695 Swedish • WT PSEN1	
#B3 H4APPSwPS1PS2KO + PS1(E280A) #21 (H4 KO APPSwe + PS1 E280A)	H4	• PSEN1 + PSEN2 Crispr/Cas KO • APP695 Swedish • PSEN1 E280A	
#B3 H4APPSwPS1PS2KO + PS2(WT) #7 (H4 KO APPSwe + WT PS2)	H4	• PSEN1 + PSEN2 Crispr/Cas KO • APP695 Swedish • WT PSEN2	
#B3 H4APPSwPS1PS2KO + PS2(N141I) #5 (H4 KO APPSwe + PS2 N141I)	H4	• PSEN1 + PSEN2 Crispr/Cas KO • APP695 Swedish • PSEN2 N141I	
HEK-Notch-CSL-Luc	HEK293	• Notch1ΔE (human Notch-1 with a deletion of the extracellular domain) • Firefly luciferase gene reporter under the control of Notch-response elements	• IMDM + 10% FBS • 50 μg/mL hygromycin • 150 μg/mL G418

Shorthand designations of cell lines (where applicable) are in parentheses in the left column and are used in the text and in figure legends for better readability.

Table 2 shows the cell lines expressing WT or mutant presenilins which were produced for this study.

**TABLE 2:** Clonal cell lines expressing wildtype or mutant presenilins.

Transfected with plasmids	Parental cell lines	
	H4 APPSwe31-10	#B3 H4APPSwe31-10 PSEN1/PSEN2 KO
pIRES-NEO2 WT PSEN1	H4 APPSwe31-10 + PS1(WT) #15	#B3H4APPSwPS1PS2KO + PS1(WT) #17
pIRES-NEO2 PSEN1 E280A	H4 APPSwe31-10 + PS1(E280A) #25	#B3H4APPSwPS1PS2KO + PS1(E280A) #21
pIRES NEO2 WT PSEN2	H4 APPSwe31-10 - PS2(WT) #3	#B3H4APPSwPS1PS2KO + PS2(WT) #7
pIRES NEO2 PSEN2 N141I	H4 APPSwe31-10 - PS2(N141l) #4	#B3H4APPSwPS1PS2KO + PS2(N141I) #5

Clonal cell lines produced by stable transfections of H4APPSwe31-10 and #B3 H4APPSwe31-10PSEN1/PSEN2 KO cell lines with plasmids encoding WT PSEN1 and PSEN1 E280A, as well as WT PSEN2 and PSEN2 N141I. See Materials and Methods for technical details of the transfection and Table 2 for cell culture media and shorthand names for the cell lines.

### 2.3 Cell culture

All cell lines used in this study were cultured in medium based on IMDM (Iscove’s Modified Dulbecco’s Medium) supplemented with 10% (v/v) heat inactivated fetal bovine serum (FBS) and the appropriate antibiotics as detailed in Table 1 using standard cell culture techniques (Freshney, 2016). Cells were generally cultured in cell culture flasks (Corning) in a humidified cell culture incubator at 37 °C with 10% (v/v) CO_2_ and passaged 1–3 times per week; HEK293 cell lines were cultured in collagen-coated flasks. All cell lines expressing APP or APPSwe were passaged with Versene (Thermo Fisher, Waltham, United States), all other cell lines were passaged with 0.05% Trypsin (Gibco, Waltham, United States). The names and properties, as well as cell culture media used for the cell lines in this study are detailed in Table 1.

### 2.4 AlphaLISA assay and data acquisition

#### 2.4.1 Measuring Aβ production in cell culture supernatants

Cells were plated in multi-well plates on the same day that compounds were added to the cells. For the different H4 cell lines used in this study, cells were plated in 100 μL at 5 × 10^4^ cells/well in a tissue culture treated 96-well plate (Falcon 353075). HEK293 APPWT cells were plated at a density of 8 × 10^4^ cells/well into 96-well plates. N2A cells were plated at a density of 15 × 10^4^ cells/well in D-lysine coated 96-well plates. Compounds were prediluted in DMSO to a 100x concentration and then further prediluted in cell culture medium to reach a 3x concentration. Then, 50 µL of the diluted compounds were added to the cells. Compounds were tested in an 8- or 11-step concentration range with semilogarithmic dilution steps. After overnight incubation (18–22 h) at 37 °C and 5% CO_2_ in a humidified cell culture incubator, the cell culture supernatants were collected and Aβ was measured using an AlphaLISA (Alpha: amplified luminescent proximity homogeneous assay) assay.

For the detection of Aβ42, AlphaLISA acceptor beads were coupled with the detection antibody AL12F4 (Covance, Princeton, United States); biotinylated 4G8 antibody was used as a secondary antibody for the detection (BioLegend, San Diego, United States). For the assay, 10 μL cell culture supernatant sample was combined with 5 µL of a 1 + 1 mixture of AL12F4-coupled acceptor beads and biotinylated 4G8 antibodies in a 384-well Optiplate (Perkin Elmer, Shelton, United States). The plate was sealed and incubated on a shaker protected from light for either 3 h at RT or overnight at 4 °C. Subsequently, 10 µL streptavidin-coupled donor beads were added, the plate was incubated for another 30 min on a shaker at RT, and the plate was read on a Paradigm AlphaLISA reader (Excitation: 680 nm, Emission: 570 nm).

The levels of Aβ42 concentrations in the culture supernatants were calculated as percentages of the maximum signal obtained from cells cultured without any treatment. The IC_50_ values were calculated using Excel XLfit software (IDBS software package, Woking, UK). A similar procedure was applied for other Aβ species with different antibody combinations as outlined in Table 3.

**TABLE 3:** Antibody combinations used for detection of Aβ fragments in cell culture supernatants by AlphaLISA.

Analyte	Capture antibody	Detection antibody
Cell culture supernatants^a^		
Aβ42	Biotin 4G8 (Biolegend, 800704)	AlphaLISA labelled 12F4 (Covance, SIG-39142–1,000)
Aβ40	Biotin 4G8 (Biolegend, 800704)	AlphaLISA labelled Bap24 (F. Hoffmann-La Roche AG^b^)
Aβ37	Biotin 13G15 (US biological, 137408)	AlphaLISA labelled 82E1 (IBL, 10323F)
Aβ38	Biotin MAK5.002.18 (F. Hoffmann-La Roche AG^b^)	AlphaLISA labelled 82E1 (IBL, 10323F)
Aβ total	Biotin 4G8 (Biolegend, 800704)	AlphaLISA labelled 6E10 (Covance, SIG-39320–1,000)
Tissue lysates^a^		
Aβ42	Bio12F4 (Biolegend, 805504)	AlphaLISA labelled 82E1 (IBL, 10323F)

The AlphaLISA assay protocol is the same for all Aβ fragment lengths as described for Aβ42 in the Materials and Methods.

^a^ For tissue lysates, the same antibody combinations are used as for cell culture supernatants, except for Aβ42.

^b^ Antibodies produced in house at F. Hoffmann-La Roche AG.

#### 2.4.2 Detection of Aβ peptides in mouse brain homogenates

Frozen brain halves without cerebellum were suspended in a ratio of 10% (w/v) in ice cold diethylamine (DEA) homogenization buffer consisting of 1% DEA and 50 mM NaCl supplemented with complete protease inhibitors (Roche Diagnostics, Rotkreuz, Switzerland) and homogenized in a MagNA Lyser Green Beads (Roche Diagnostics, Rotkreuz, Switzerland) by two 20-s pulses in Precellys (Bertin Technologies, Montigny-le-Bretonneux, France) at 6,500 rpm at RT. After 3 h incubation on ice, homogenates were centrifuged at 100,000 × g for 60 min at 4 °C. After centrifugation, supernatants were collected and stored at −80 °C for further processing.

Homogenates in DEA were neutralized with 0.025 M citric acid in H_2_O and then diluted in assay buffer (AlphaLISA HiBlock Buffer, Revvity, Waltham, United States). The optimal dilution range depended on the analyzed brain tissue and was tested in pilot experiments to ensure that the assay was performed within its dynamic range.

The analysis of brain homogenates with AlphaLISA was performed essentially as described for cell culture supernatants with minor modifications. For the detection of Aβ42, AlphaLISA acceptor beads were coupled with the detection antibody AL82E1 (IBL 10323F, Männedorf, Switzerland); biotinylated 12F4 antibody was used as secondary antibody for the detection (BioLegend 805504, San Diego, United States). The antibody combinations used for detection of other Aβ species in brain lysates are outlined in Table 3.

For the assay, 5 µL of neutralized and diluted DEA brain extract sample or standard were combined with 5 µL of a 1 + 1 mixture of AL82E1-coupled acceptor beads and biotinylated 12F4 antibodies in a 384-well Optiplate (Perkin Elmer, Shelton, United States). The plate was sealed and incubated for 3 h on a shaker at RT protected from light. Subsequently, 15 µL streptavidin-coupled donor beads were added, the plate was incubated for another 30 min on a shaker at RT, and the plate was read on a Paradigm AlphaLISA reader (Excitation: 680nm, Emission: 570 nm). Aβ standards were diluted in a mixture of 1 + 1 DEA buffer + 0.025 M citric acid + 12% heat inactivated FBS.

### 2.5 Notch-1 selectivity assay

The HEK-Notch-CLS-Luc cells were cultivated as described above, and the assay was performed essentially as described by the manufacturer of the cell line. For each experiment, cells were plated at a density of 3.5 × 10^4^ cells/well in 100 μL in a 96-well assay plate and grown overnight. Due to lack of a control cell line, an additional plate was prepared in parallel to test for compound effects on cell viability and cell number using an ATP-based cell viability assay (CellTiter-Glo Assay, see below).

Then, semi-logarithmic dilutions of the compounds were prepared in DMSO, and the compound concentrations were adjusted to a 100x concentration. Compound solutions were further prediluted 1:33 in cell culture medium to reach a 3× concentration.

The starting concentration was 10 μM for nivegacetor and 1 μM for the GSI reference compounds DBZ and RO4929097, respectively. Control wells with the reference compound, without compound, and with medium only were included on each plate to correct for background signal and normalize the effect size to percent of control. Then, 50 μL of the diluted compounds were added to the cells. After 18–20 h incubation in the cell culture incubator at 37 °C, for detection of Notch dependent luciferase activity the cell culture medium was removed and a mixture of 75 μL of fresh medium + 75 μL Steady-Glo-Reagent (Promega, Madison, United States) was added. For measuring ATP-dependent cell viability in the control plate, the cell culture medium was removed, and 75 μL of fresh medium and 75 μL of CellTiter-Glo-Reagent (Promega G7572) were added. The plates were incubated at RT for 15–20 min on a horizontal shaker to facilitate cell lysis. Luminescence was then measured in a SpectraMax Paradigm plate reader (Molecular Devices, San Jose, United States) with the use of a luminescence cartridge.

Data calculation included a subtraction of the blank wells to account for assay background, and normalization for possible compound effects on cell number/viability measured with the CellTiter-Glo-Reagent.

In the presence of a GSIs we observed a minimum baseline luciferase signal of about 20% at GSI concentrations which completely abolish Aβ production measured in the H4 APPSwe or the HEK293 APPWT cell lines. This baseline luciferase signal was observed with multiple different GSIs. Based on this observation we attribute the baseline signal to some degree of leakiness of the reporter construct, as it is sometimes observed with gene reporter constructs (Lopreside et al., 2019).

### 2.6 Broad selectivity profiling

The selectivity of nivegacetor was tested for a range of targets composed of receptors, enzymes, transporters, and ion channels using a combination of radioligand and functional assays. The testing was initially performed at a single concentration followed up by testing in a dose-response for targets of special interests and for those targets where testing at a single concentration revealed a >50% activity at 10 μM.

The selectivity profiling was performed at Eurofins/Cerep SA (Celle Lévescault, France; part of Eurofins) and Eurofins Taiwan (New Taipei City, Taiwan). Detailed descriptions of the methods employed are described at www.eurofinsdiscoveryservices.com.

### 2.7 APPSwe transgenic mice, *in vivo* experiments

For *in vivo* profiling of nivegacetor with respect to GSM effects a transgenic mouse line was used expressing the 751 amino acid variant of the human APP protein with the ‘Swedish’ mutation (double mutation K670N, M671L) under the control of the Thy1.2 minigene (Richards et al., 2003). In brief, the cDNA sequence encoding the APP751 (X06989) was cloned and subjected to site-directed mutagenesis, introducing the ‘Swedish’ double mutation, and inserted into an expression vector comprising the mouse Thy1.2 promotor. Transgenic mice were produced with this construct using standard protocols (Brigid Hogan et al., 1994). Vector-free linear DNA comprising the APPSwe cDNA and mouse Thy1.2 minigene construct was microinjected into male pronuclei of B6D2F1 (i.e., C57BL/6J × DBA/2 F1 hybrid) zygotes. Viable zygotes were inserted into the oviducts of pseudopregnant B6D2F1 female mice, and genomic insertion of the DNA constructs were confirmed by quantitative Southern Blots performed on genomic DNA extracted from tail biopsies of the offspring. Founder animals were expanded, and the suitable line for experiments was selected based on transgene expression; the genomic insertion of the transgene was mapped to chromosome 16 with ca. 75 copy numbers of the construct inserted into the genome. The resulting line (B6; D2-Tg(Thy1-APPSwe)72Blt/Crl) dubbed APPSwe was backcrossed for 3 generations with C57BL/6J mice and maintained on a partially mixed genetic background. The transgenic mouse line was established at F. Hoffmann-La Roche AG in Switzerland and maintained in active breeding until 2016 when the colony was transferred to Charles River (France) and Taconic (Denmark).

Experiments were performed using heterozygous female mice that were produced by crossing heterozygous male mice with C57BL/6J WT females. Sixty APPSwe heterozygous female mice (N = 36 at 5 months; N = 21 at 4 months) were group housed in holding rooms at controlled temperature and humidity under a 12-h light/dark cycle with the light period starting at 6:00 a.m., and with free access to food and drinking water.

Nivegacetor and the GSI MRK-560 used as reference were formulated *ad hoc* in a vehicle composed of 5% (v/v) ethanol und 10% (v/v) solutol in water and administered by oral gavage with a dosing volume of 10 mL/kg.

For the nivegacetor dose-response experiment, 5-month-old mice were administered either vehicle (control), nivegacetor at 1, 3, 10, 30 mg/kg, or the GSI MRK-560 as positive control at 30 mg/kg (N = 6 per group). Treatment groups were pseudo-randomized by systematic alternation of treatments (A–B – C–D – E, A–B – C–D – E, *etc.*), and the experimenters were blinded to the assignment of treatments to individual animals. Four hours later, animals received a terminal anesthesia of 150 mg/kg pentobarbital diluted with physiological NaCl solution i.p. before sacrifice and collection of brain and plasma which were stored frozen at −80 °C until further processing.

For the nivegacetor time course experiment, 4-month-old mice were treated with nivegacetor at 25 mg/kg and were sacrificed at the following time intervals post dosing: 1 h; 2.5 h; 4 h; 7 h; 10 h; 16 h; 24 h (N = 2–3/group) following the procedure described above. A baseline (control) group of mice was sacrificed prior to dosing (N = 4). Also for the time-course experiments, treatment groups were pseudo-randomized by systematic alternation of treatments, and experimenters were blinded to the assignment of treatments to individual animals.

For both experiments, the full spectrum of Aβ peptides (Aβ37, Aβ38, Aβ40, Aβ42, and Aβ total) was profiled in soluble DEA brain extracts, as described in Section 2.4.2. Aβ levels were expressed as a percentage of the vehicle group in the dose-response experiment, and as a percentage of the baseline group for the time course experiment. Sample sizes were based on historical data within the laboratory. No criteria were set for excluding data points during the analysis. All data were included in the analysis by one-way ANOVA, followed by *post hoc* Dunnett’s comparison test (Graphpad Prism, Boston, United States). Statistical significance was predetermined as p<0.05.

### 2.8 Preparation of frozen tissue sections from an individual with Alzheimer’s disease and autoradiography

Brain tissue from an individual with AD was obtained from the Sun Health Research Institute (Sun City, Arizona). The brain tissue was collected within the frame of a donation program under written consent between 1.5 and 2.5 h after the patient’s death and immediately frozen at −80 °C. Neuropathological staging was performed based on the occurrence of neurofibrillary tangles, according to Braak. Frontal cortex tissue from an 80-year-old female individual with Braak VI stage showing a high incidence of amyloid plaques was used for this study.

The brain tissue block was cut in 10 μm thick sections using a cryostat (Leica Biosystems, Nussloch, Germany) at −17 °C chamber temperature and −15 °C object temperature. Sections were transferred to Histobond+ microscope slides (Marienfeld Laboratory Glassware; Lauda-Königshofen, Germany). After drying for 2 h at RT, the sections were stored at −20 °C until further use.

The frozen brain tissue sections were thawed and air dried at RT for 15 min. Brain tissue sections were directly incubated with [^3^H]florbetaben at 1.7 nM for the first experiment and with 2.1 nM for the second experiment. Incubation was performed in phosphate-buffered saline (PBS) containing 0.1% bovine serum albumin in the presence or absence of nivegacetor (10 μM or 100 nM) or florbetapir (10 μM) at RT for 60 min. After washing twice for 5 min at 4 °C in PBS and 3 quick dips in distilled water at 4 °C, the sections were dried in a cold stream of air at 4 °C for 2 h. Subsequently, the sections were placed in a FujiFilm Cassette (Cytiva, Marlborough, United States) together with a tritium microscale standard (American Radiochemicals, Saint Louis, United States) and exposed to a FujiFilm Imaging Plate (Cytiva, Marlborough, United States) for 5 days.

After the exposure, the Storage Phosphor Screens were scanned using a BAS-5000 FujiFilm Imaging Plate Reader (Cytiva, Marlborough, United States) at a resolution of 25 μm/pixel. Visualization and quantification of the autoradiograms was performed using the MCID analysis software (version 7.0; Vision Systems Design, St. Catharines, Canada).

Quantification of radioligand binding was performed by a region of interest (ROI) analysis of autoradiograms. White and grey matter were delineated manually on the autoradiograms, and the amount of [^3^H]florbetaben bound to the grey matter of the tissue section was expressed as fmol of [^3^H]florbetaben/mg of protein (fmol/mg protein).

To assess the percentage of tracer displacement in the AD tissue, the specific binding (SB) was calculated as follows:

SB [^3^H]florbetaben = [^3^H]florbetaben binding in grey matter - [^3^H]florbetaben binding in white matter.

The percentage of displacement was calculated from:

$$\%\text{ displacement}=100-(\frac{100}{\text{SB}\;\left(\left[{\text{​}}^{3}H\right]\text{florbetaben}\right)}\times \text{SB }\left(\left[{\text{​}}^{3}H\right]\text{florbetaben with blocker}\right))$$

The % change of the [^3^H]florbetaben binding in the absence (total binding) or presence of nivegacetor and florbetapir, respectively, was calculated with normalization to total binding = 100%.

## 3 Results

### 3.1 Nivegacetor is a potent gamma secretase modulator

**FIGURE 3:**
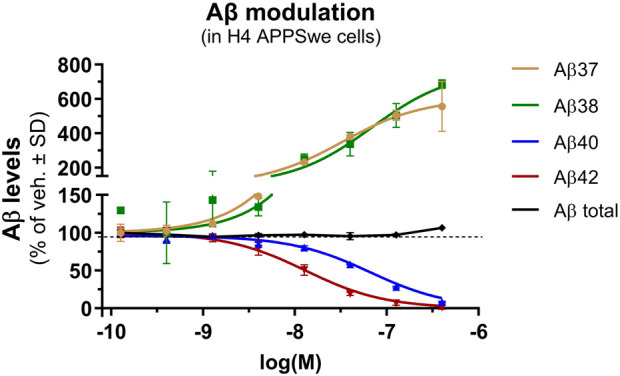
Effect of nivegacetor on the production of Aβ fragments of different lengths, measured in H4 APPSwe cells. The cells were incubated overnight with vehicle or nivegacetor, and Aβ fragments were quantified in the cell culture supernatant. Changes in Aβ levels are expressed as percent of Aβ levels in vehicle-treated H4 APPSwe cells. Data is plotted as mean of N = two to five replicates ± S.D.

Nivegacetor was characterized with respect to its ability to modulate the length of Aβ peptides *in vitro* using cell lines overexpressing human APP in its wild-type form or with the Swedish mutation K670N/M671L (APPSwe) (Haass et al., 1995). Nivegacetor dose-dependently reduced the production of Aβ42 and Aβ40 in H4 cells expressing APPSwe (H4 APPSwe) while concomitantly increasing the production of Aβ37 and Aβ38 (Figure 3). The potency of nivegacetor for lowering Aβ42 in this cell line is IC_50_ = 12.4 ± 1.8 nM, without correcting for compound binding to the 10% (v/v) heat-inactivated bovine serum that is present in the cell culture medium. The production of total Aβ (comprising Aβ42, Aβ40, Aβ37, and Aβ38), measured with an antibody detecting the N-terminal domain of Aβ, remained unchanged in the presence of nivegacetor, indicating that the gamma secretase enzyme activity is not inhibited by nivegacetor.

In comparison to H4 cells expressing APPSwe, nivegacetor showed a slightly higher Aβ42 lowering potency in HEK293 cells expressing WT human APP (IC_50_ = 8.4 ± 4.8 nM) and in the murine neuroblastoma cell line N2A expressing APPSwe (IC_50_ = 7.8 ± 1.9 nM).

### 3.2 Nivegacetor does not inhibit Notch-1 and is selective over a broad range of drug targets

The *in vitro* selectivity of nivegacetor for modulating the processing of APP over other gamma secretase substrates was tested on Notch-1 as a representative target, given that gamma secretase is known to process multiple other substrates with important physiological functions (Medoro et al., 2018).

**FIGURE 4:**
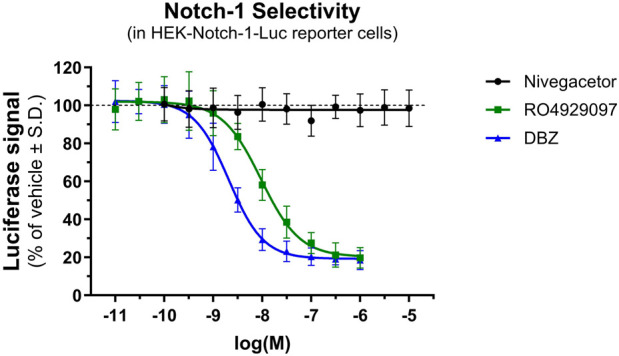
Nivegacetor does not inhibit gamma secretase. Nivegacetor was tested in dose-response in a Notch-1 gene reporter assay in HEK293 cells, using the potent gamma secretase inhibitors RO4929097 and DBZ as positive controls. The percent change of luciferase signal compared to vehicle (DMSO), normalized to compound effects on cell viability, is depicted as mean ± SD. Each point represents N = 10 replicates for nivegacetor, N = 8 replicates for RO4929097, and N = 10 replicates for DBZ.

Nivegacetor was tested in a cell-based Notch-1 gene reporter assay in which the cleaved Notch-1 C-terminal fragment drives expression of a luciferase reporter. Nivegacetor did not inhibit Notch-1 processing when tested up to a concentration of 10 μM (Figure 4). In contrast, the potent GSIs RO4929097 (Luistro et al., 2009) and DBZ (Milano et al., 2004) inhibited Notch-1 signaling in a dose-dependent manner.

Furthermore, nivegacetor was extensively profiled with respect to its pharmacological selectivity in 138 different assay covering a wide range of targets unrelated to gamma secretase, employing a panel of *in vitro* assays with targets including receptors, ion channels, and enzymes. Nivegacetor was tested in these assays at a single concentration of 10 μM (Supplementary Table S1). As a follow-up, nivegacetor was tested in dose-response in a small set of radioligand binding and functional assays which included those targets on which nivegacetor showed ≥50% activity at 10 μM (Table 4).

**TABLE 4:** Pharmacological activity of nivegacetor tested in dose-response in selected assays, including targets with ≥50% activity at 10 μM.

Target	Activity at 10 μM^a^ (% control inhibition)	Dose-response follow-up (IC_50_ in μM)
Adenosine A3 receptor (hu)	16.4^b*^	>30^c^
Adrenergic α2A receptor (hu)	5.6^b^	8.8^c^
GABA-A (non-selective)	65.7^b^	6.9
Matrix metalloprotease 24 (hu)	35.5^c^	>10^c^
Muscarinic receptor 1 (hu)	40.9^b^	10^c^
Muscarinic receptor 2 (hu)	49.1^b^	>30^c^
Serotonin receptor 5-HT1A (hu)	69.3^b*^	>30^c^
Serotonin receptor 5-HT2B (hu)	40.7^b*^	25^c^
Sigma receptor (non-selective) (hu)	66.7^b*^	16.6^b*^
Platelet-activating factor receptor (hu)	59.6^b*^	7.4^b*^

Nivegacetor was tested in dose-response up to concentrations of 10 or 30 μM on the selected targets. Data is expressed as change of % control, with 0 the lowest and 100 the maximal effect. Numerically negative results in tests at a single concentration are interpreted as a reflection of assay variability and lack of activity on the given targets.

^a^ The full list of single-concentration selectivity data for nivegacetor is summarized in Supplementary Table S1. All dose-response experiments went up to a concentration of 30 μM except matrix metalloprotease 24 which was tested up to 10 μM.

^b^ Radioligand binding experiments; assays employing agonist radiotracers are indicated by an asterisk (*). hu: human.

^c^ Functional (cell-based or enzymatic) assays configured for detection of antagonist activities were used.

Overall, these studies revealed that nivegacetor is more than 500-fold selective for gamma secretase over all other targets tested.

### 3.3 Nivegacetor modulates Aβ *in vivo* in a transgenic mouse line expressing human APP with the ‘Swedish’ mutation

Nivegacetor was tested for its effect on soluble Aβ levels in brain tissue of APPSwe transgenic mice. These mice produce elevated levels of human Aβ peptides and develop a progressing amyloidosis; however, at ages of less than 5 months this mouse line can be used to study drug effects on soluble Aβ levels in the absence of aggregated Aβ deposits.

**FIGURE 5:**
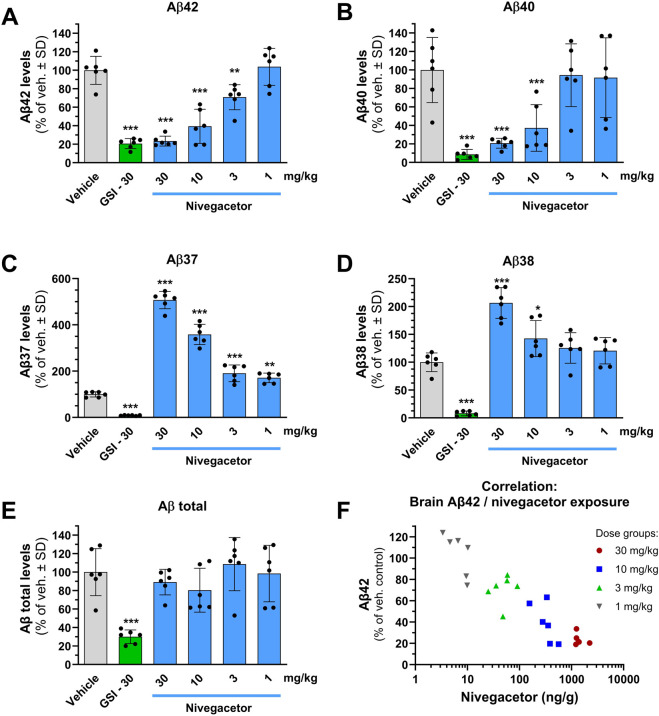
Dose-dependent modulation of soluble Aβ levels in brain tissue of APPSwe transgenic mice. The levels of Aβ42 **(A)**, Aβ40 **(B)**, Aβ37 **(C)**, Aβ38 **(D)**, and total Aβ **(E)** in soluble DEA brain extracts of 5 months old female APPSwe transgenic mice after single oral doses of nivegacetor are shown. The brain drug exposure was measured and correlated with the Aβ42 levels in brain extracts **(F)**. Each group consists of N = 6 animals, Aβ levels are depicted as percent of Aβ levels in brain extracts from vehicle-treated animals. GSI = MRK-560. Statistical testing by one-way ANOVA, followed by *post hoc* Dunnett’s comparison test: ** p < 0.01; *** p < 0.001.

Nivegacetor was dosed orally as a microsuspension in a dose-range of 1–30 mg/kg along with the GSI MRK-560 at 30 mg/kg as a positive control, and Aβ levels were measured 4 h after dosing by AlphaLISA in soluble DEA brain extracts. Nivegacetor dose-dependently reduced Aβ42 levels in brain tissue (Figure 5A; Table 5), with a 77% reduction at a dose of 30 mg/kg, a 61% reduction at 10 mg/kg, and a 29% reduction at 3 mg/kg. Moreover, nivegacetor dose-dependently reduced Aβ40 levels (Figure 5B) and concomitantly increased Aβ37 (Figure 5C) and Aβ38 (Figure 5D) levels in brain tissue without altering the total amount of Aβ peptides (Figure 5E). In contrast to nivegacetor, the GSI MRK-560 reduced the levels of all Aβ species measured. The dose-response relationship of nivegacetor lowering Aβ42 in brain tissue is apparent from the correlation of drug exposure and Aβ42 levels in brain tissue (Figure 5F).

**TABLE 5:** Dose-dependent modulation of Aβ levels by nivegacetor in brain tissue of APPSwe transgenic mice.

​	Vehicle	GSI	Nivegacetor			
		30 mg/kg	30 mg/kg	10 mg/kg	3 mg/kg	1 mg/kg
Aβ42 (%)	100.0 ± 15.2	20.7 ± 5.4	23.4 ± 5.3	39.4 ± 18.4	70.8 ± 13.6	103.7 ± 20.0
Aβ40 (%)	100.0 ± 35.1	8.6 ± 5.4	20.8 ± 5.3	37.3 ± 25.2	94.3 ± 33.9	91.6 ± 43.1
Aβ38 (%)	100.0 ± 16.7	8.1 ± 4.2	206.5 ± 27.6	142.5 ± 32.4	125.5 ± 27.3	120.6 ± 23.7
Aβ37 (%)	100.0 ± 11.4	8.6 ± 1.5	507.2 ± 37.5	358.4 ± 43.7	190.7 ± 35.9	171.1 ± 20.8
Aβ total (%)	100.0 ± 25.4	30.0 ± 7.4	89.2 ± 13.8	80.4 ± 23.7	108.6 ± 28.7	98.4 ± 30.5
Exposure (ng/g)	−	n.a	1,445.0 ± 391.3	346.1 ± 134.6	52.6 ± 22.9	7.3 ± 2.9

Changes of soluble Aβ levels measured in DEA brain extracts of 5 months old female APPSwe transgenic mice after single oral doses of nivegacetor, along with the mean total drug exposure in brain tissue per dose group (data plotted in Figure 5). Each group consists of N = 6 animals. Aβ levels are depicted as percent of Aβ levels in brain extracts from vehicle-treated animals. Nivegacetor brain tissue concentrations (Exposure) are given as total drug concentration in ng nivegacetor/g brain tissue. All data are depicted as mean ± S.D. n.a., not analyzed.

**FIGURE 6:**
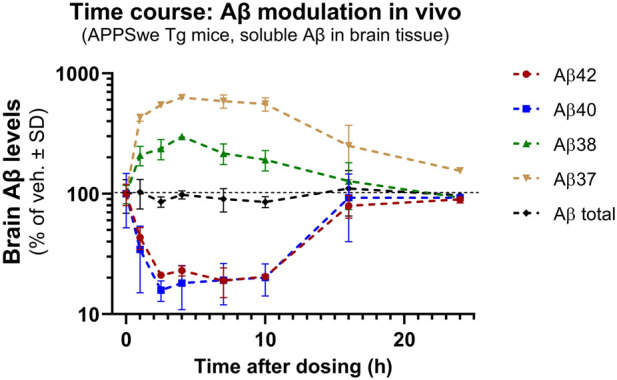
Time course of gamma secretase modulator effects on the spectrum of Aβ peptides in APPSwe transgenic mice after a single oral dose of 25 mg/kg of nivegacetor. Nivegacetor-treated APPSwe transgenic mice were sacrificed at the indicated time intervals post dosing and the levels of Aβ peptides were measured in soluble DEA brain extracts. Aβ levels are displayed normalized as percent of the vehicle levels. Each group consists of N = 3 animals. Data are displayed as mean ± SD; note the logarithmic scale of the Y-axis.

In order to further characterize the GSM effects on the Aβ profile in brain tissue *in vivo*, 4 months old APPSwe mice were dosed with a single oral dose of 25 mg/kg nivegacetor, and the full spectrum of Aβ peptides (Aβ37, Aβ38, Aβ40, Aβ42, and Aβ total) was profiled in soluble brain extracts after different time intervals post dosing. Here a robust reduction of Aβ42 and Aβ40 of up to 80% from about 2 to 10 h post dosing was observed. A robust concomitant increase in Aβ37 and Aβ38 levels was observed which parallels the pattern observed *in vitro*, indicating a good *in vitro* to *in vivo* correlation of the GSM effect. The levels of total Aβ (probed with an antibody recognizing the N-terminus of Aβ) remained unchanged, indicating the lack of gamma secretase inhibition (Figure 6).

### 3.4 Nivegacetor is active and equipotent on the PSEN1 E280A ‘Columbian’ autosomal dominant Alzheimer’s disease mutation

The possible impact of two well-known ADAD mutations, the PSEN1 E280 ‘Columbian’ mutation (Lalli et al., 2014) and the PSEN2 N141I ‘Volga German’ mutation (Tomita et al., 1997) on the potency of nivegacetor was studied.

For these experiments the WT forms of PSEN1 and PSEN2, or the mutated isoforms were stably transfected into H4 APPSwe cells with or without prior targeted disruption of both endogenous PSEN1 and PSEN2 genes, respectively (Tables 1, 2). Even though it is known that simple overexpression of mutated forms of presenilins dominate the function of the gamma secretase in cell lines with the endogenous WT forms of presenilins intact (De Strooper et al., 2012; Heilig et al., 2013), we included experiments with a double knock-out of the endogenous WT PSEN1 and PSEN2 genes to generate a situation where the overexpressed mutated and WT presenilins studied were the sole isoforms expressed.

**FIGURE 7:**
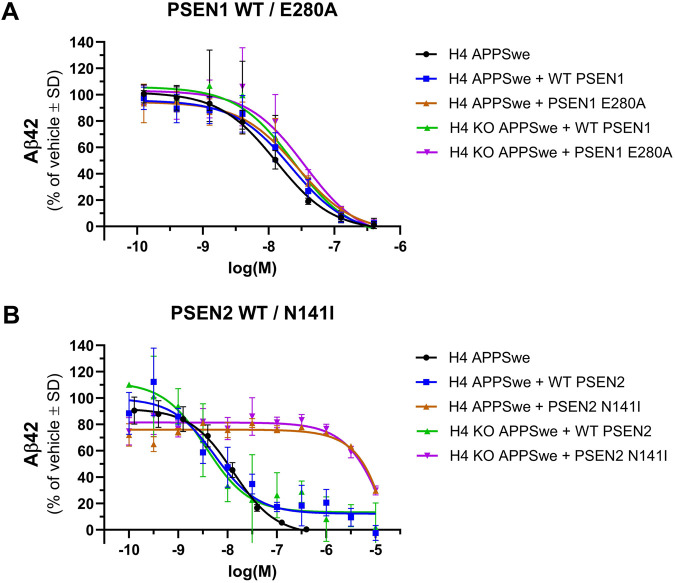
Aβ42 lowering potency of nivegacetor in the presence or absence of ADAD mutations PSEN1 E280A and PSEN2 N141I. Nivegacetor was tested for its Aβ42 lowering capability in the presence or absence of presenilin mutations, by comparing the potency of nivegacetor measured in H4 APPSwe cells and in H4 APPSwe cells with a targeted disruption of both PSEN1 and PSEN2 genes, transfected with **(A)** WT PSEN1 and PSEN1 E280A, and with **(B)** WT PSEN2 and PSEN2 N141I, respectively. For each transfection, a monoclonal stably transfected cell line was generated. Each datapoint represents the mean ± SD of N = 3-7 independent replicates.

The experiments with the mutated form of PSEN1 revealed that nivegacetor showed a similar Aβ42 lowering potency in the cell lines generated with intact endogenous presenilin genes (WT PSEN1 and PSEN1 E280A), and with the double PSEN1/PSEN2 knock-out (WT PSEN1 and PSEN1 E280A) (Figure 7A; Table 6).

**TABLE 6:** Effect of ADAD mutations PSEN1 E280A and PSEN2 N141I on the Aβ42 lowering potency of nivegacetor.

​	WT H4	WT PSEN1		PSEN1 E280A		WT PSEN2		PSEN2 N141I	
​	H4 APPSwe	H4 APPSwe + WT PSEN1	H4 KO APPSwe + WT PSEN1	H4 APPSwe + PSEN1 E280A	H4 KO APPSwe + PSEN1 E280A	H4 APPSwe + WT PSEN2	H4 KO APPSwe + WT PSEN2	H4 APPSwe + PSEN2 N141I	H4 KO APPSwe + PSEN2 N141I
Mean IC_50_ ± SD (nM)	16.98 ± 5.43	17.40 ± 8.08	21.86 ± 10.39	20.45 ± 8.20	27.78 ± 12.87	5.66 ± 1.42	5.11 ± 3.65	4325.79 ± 2,532.80	6,030.80 ± 4456.43
N	7	5	4	5	4	3	3	3	3

The effect of each mutation was tested in H4 APPSwe cells, and in H4 APPSwe cells with homozygous targeted disruption of PSEN1 and PSEN2 genes (H4 KO APPSwe), respectively. For each WT or mutated isoform of PSEN1 and PSEN2, clonal stably transfected cell lines were produced. Cells were incubated overnight with nivegacetor in a dose-response in presence of 10% FBS, and Aβ42 content in the conditioned medium was measured by AlphaLISA. N: Number of independent experiments with 2-4 technical replicates per experiment; the mean ± SD is presented.

In contrast, the experiments with the PSEN2 N141I mutations resulted in a profound loss of potency regardless of whether the mutated presenilin was expressed in the H4 APPSwe cell line with the intact endogenous presenilin genes (WT PSEN2 and PSEN2 N141I), or with the double PSEN1/PSEN2 knock-out (WT PSEN2 and PSEN2 N141I) (Figure 7B; Table 6).

We noticed that the overexpression of WT PSEN1 caused only minimal changes in the Aβ42 lowering potency of nivegacetor which are well within the normal range of assay variability, while overexpression of WT PSEN2 led to an increased potency compared to the untransfected H4 APPSwe cells (Table 6).

### 3.5 Nivegacetor does not interfere with the amyloid PET tracer florbetaben

Nivegacetor addresses the production of amyloidogenic Aβ and ultimately the buildup of aggregated Aβ deposits in the brain which, in human, is monitored with methods such as positron emission tomography (PET) imaging. Consequently, it is relevant to probe whether nivegacetor might interfere with the binding of amyloid PET tracers to aggregated Aβ. [^18^F]Florbetaben is an established PET ligand which is used in clinical trials to assess Aβ plaque burden in the brains of patients with AD (Sabri et al., 2015). In this study we used [^3^H]florbetaben to probe binding of the tracer on human AD brain sections in the absence or presence of nivegacetor and another established Aβ PET tracer, florbetapir (Wong et al., 2010).

**FIGURE 8:**
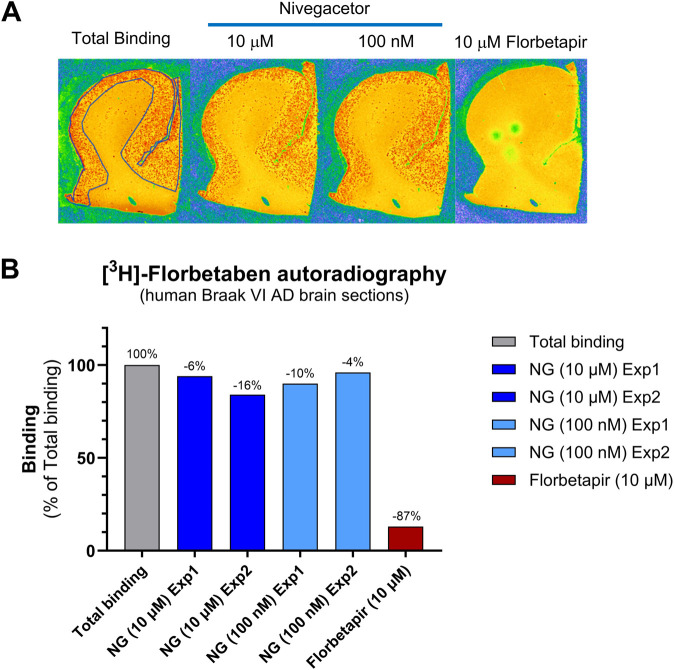
Autoradiography images of human Braak VI Alzheimer’s Disease brain tissue sections from cortex, co-incubated with the tracer [^3^H]florbetaben and the cold compounds nivegacetor or florbetapir. **(A)** Autoradiography of a human cortical brain tissue section from a Braak VI AD patient incubated with [^3^H]florbetaben in the absence or presence of nivegacetor and florbetapir, respectively. The grey matter in the tissue section is delineated manually and indicated by a blue line. The amount of bound radioactivity is displayed color-coded, with no/low intensity in blue, and high intensity in red/yellow. **(B)** Quantification of [^3^H]florbetaben binding to the tissue sections in absence or presence of nivegacetor or florbetapir, normalized to total binding = 100%. Nivegacetor was tested in two independent experiments, Exp. 1 and Exp.2. NG: Nivegacetor.

Strong binding of [^3^H]florbetaben (at approximately 2 nM) to Aβ plaque pathology was observed in the grey matter of a Braak VI cortical tissue section in the absence of any blocker (Figure 8). In the presence of nivegacetor at a concentration of 100 nM or 10 μM radioligand binding was not reduced and the characteristic punctate binding pattern remained. The established Aβ ligand florbetapir was used as a positive control at a concentration of 10 μM and almost completely blocked [^3^H]florbetaben binding to Aβ plaques, with some residual non-specific or background binding of the radioligand. The data demonstrate that nivegacetor does not interact with the [^3^H]florbetaben binding site in Aβ amyloid plaques.

## 4 Discussion

Here we describe the properties of nivegacetor, a novel GSM that potently reduces the production of Aβ42 and Aβ40 while concomitantly increasing the levels of Aβ37 and Aβ38, in line with the expected molecular mechanism of action for a GSM (Figure 1). While the individual Aβ levels changed, the amount of total Aβ produced across different fragment lengths remained unchanged, indicating that nivegacetor does not inhibit gamma secretase. The absence of gamma secretase inhibition was also confirmed for Notch-1 as a representative gamma secretase substrate unrelated to APP. The selectivity profile of nivegacetor for targets other than gamma secretase was studied by testing the compound in 138 assays on a range of diverse targets including receptors, enzymes, transporters, and ion channels, employing a mix of radioligand binding and functional assays. The profiling includes two key enzymes involved in the processing of APP, BACE-1 and matrix metalloprotease 24 (MMP24) also known as eta-secretase (Willem et al., 2015; Baranger et al., 2016) which cleaves APP far N-terminal to the BACE-1 site. A recent study demonstrated that a specific cleavage fragment, AETA, modulates NMDAR currents and synaptic spine morphology (Dunot et al., 2024) underscoring the physiological relevance of the eta-secretase pathway, prompting us to specifically test nivegacetor for a possible activity on this pathway. We confirmed the selectivity of nivegacetor over MMP24 (Table 4; IC_50_ >10 μM), which is in line with the absence of significant activity on the other matrix metalloproteases 1, 2, and 9 (Supplementary Table S1). Taken together, these studies revealed a more than 500-fold selectivity of nivegacetor when comparing the Aβ42 lowering activity to the studied off-target panel.

Nivegacetor was also effective in lowering Aβ42 and Aβ40 levels when dosed orally in transgenic mice expressing the human APP protein with the ‘Swedish’ K670N/M671L double mutation; the Swedish APP variant leads to an increased rate of Aβ production compared to WT APP (Citron et al., 1992). In the current study, nivegacetor effectively lowered Aβ42 with a maximal effect of −77% at a dose of 30 mg/kg at 4 h after dosing compared to the vehicle group (Figure 5A). In this dose-response experiment, levels of soluble Aβ42 and nivegacetor concentrations in brain tissue showed a good correlation (Figure 5F). When recording a time-course of Aβ modulation in brain tissue of the same transgenic mouse line following a dose of 25 mg/kg, the same pattern of Aβ42 and Aβ40 lowering and concomitant Aβ38 and Aβ37 increase was apparent (Figure 6), which was also observed *in vitro* (Figure 3). The 4 h pretreatment time interval was chosen to accommodate the time required for drug absorption, gamma secretase modulation, and the modulated Aβ profile in brain tissue to reach a new equilibrium, which is driven by the *de novo* production and clearance of Aβ peptides. As seen in Figure 6, the maximum drug effect for all Aβ isoforms is reached at 4 h after the single oral dose of nivegacetor.

Nivegacetor was studied on two representative ADAD mutations, the PSEN1 E280A (Columbian) and the PSEN2 N141I (Volga German) mutation since some mutations have been reported to render GSMs inactive or lower their potency (Kretner et al., 2011; Trambauer et al., 2025). The data reveal that the presence of the PSEN1 E280A mutation has essentially no effects on the potency of nivegacetor, while the presence of the PSEN2 N141I mutation causes a more than 100-fold lowering of nivegacetor’s potency. These data are in line with reports of similar observations with different GSMs on the same mutations (Kretner et al., 2011; Lindemann et al., 2024).

The exact molecular mechanisms by which mutations in presenilins have either no effects or cause significant shifts in the potency of a GSM are not well understood. Based on the available Cryo-EM structures of the gamma secretase complex with the known binding pockets for GSMs and for the APP substrate (Yang et al., 2021) we can derive that the PSEN1 E280A mutation is located more distal to the GSM binding pocket compared to the PSEN2 N141I mutation (Supplementary Figure S2). Understanding the molecular mechanisms by which ADAD mutations impact the pharmacology of GSMs would require dedicated studies involving site-directed mutagenesis, biochemistry, and work on the gamma secretase structure in the presence of the mutations. The key takeaway of these experiments is that ADAD mutations can impact the activity and potency of GSMs which can have implications on the required drug doses for therapeutic use in ADAD mutation carriers. Since the presence or absence of such effects and their magnitude cannot be reliably predicted *in silico*, these effects require experiments for each mutation and for each GSM in question.

An unexpected observation in these experiments was that overexpression of WT PSEN2 led to an apparent increase in the potency of nivegacetor by approximately 3-fold compared to untransfected H4 APPSwe cells and to H4 APPSwe cells with WT PSEN1 overexpression, respectively (Table 6). Studies in human iPS-derived neurons with conditional knock-outs of either PSEN1 or PSEN2 revealed that gamma secretase complex comprising either PSEN1 or PSEN2 are effective in processing APP (Watanabe et al., 2021). Given that PSEN1 and PSEN2 are homologues with only about 67% amino acid sequence identity and that GSMs bind to the gamma secretase complex by direct interaction with the presenilin moiety (Ebke et al., 2011; Yang et al., 2021), it is conceivable that differences in the amino acids lining the GSM binding pocket might underlie the apparent potency differences.

Lastly, we examined whether nivegacetor has effects on [^3^H]florbetaben, the tritiated version of the amyloid PET tracer [^18^F]florbetaben, binding to Aβ plaque pathology in human AD brain tissue sections. This is an important aspect given that the most proximal effects of nivegacetor in therapeutic use are related to Aβ plaque depositions which are quantified in human by PET tracers such as [^18^F]florbetaben. The autoradiography experiments revealed that nivegacetor does not interfere with [^3^H]florbetaben binding to Aβ plaque pathology, confirming that this PET tracer can be used in the presence of nivegacetor.

Nivegacetor has not been examined yet in chronic studies in transgenic mice with amyloidosis given that single doses of the compound in mice provide less than 24 h coverage (Figure 6) which poses a challenge for undertaking long-term studies. However, studies with other compounds indicate that chronic GSM treatment in mice can slow or halt Aβ plaque buildup (Rogers et al., 2012; Brendel et al., 2015; Nordvall et al., 2025) and reduce microglial inflammation markers such as GFAP and CD11b (Rogers et al., 2012). Moreover, studies in 3D cell cultures with human iPS-derived neurons suggest that GSM treatment may reduce markers such as Tau p-Thr181 (Wagner et al., 2017).

There have been substantial drug discovery efforts in the field of GSMs by many laboratories and pharmaceutical companies which resulted in optimized brain penetrable molecules, some of which were advanced to clinical testing in healthy subjects, including BMS-932481 (Soares et al., 2016), PF-06648671 (Ahn et al., 2020), and E2212 (Yu et al., 2014). However, these compounds have not been progressed to studies in individuals with AD. Another GSM, EVP-0962, has been studied preclinically (Rogers et al., 2012) and advanced into a Phase 1 study with healthy volunteers as well as individuals with mild cognitive impairment or early AD [NCT01661673 (Hur, 2022)]; the trial is registered as completed at Clincialtrials.gov with the latest update from January 2014, but no results have been published. In addition, there are reports of multiple GSMs at various stages of preclinical development for which no clinical development in individuals with AD has been reported up to now.

An unusual prototypical GSM worth discussing is tarenflurbil, the R-enantiomer of the non-steroidal anti-inflammatory drug (NSAID) flurbiprofen, a carboxylic acid. Tarenflurbil shows modest GSM efficacy preclinically, selectively lowering Aβ42 with an *in vitro* potency of approximately 100 μM (Eriksen et al., 2003) without modulating Aβ40. Tarenflurbil has been studied clinically in a Phase 1 study in healthy elderly individuals with 3 weeks of dosing (Galasko et al., 2007) where it showed good tolerability, but only modest effects on Aβ42 plasma levels and no changes in CSF Aβ42 levels. Tarenflurbil was subsequently tested in a Phase 2 study in individuals with mild AD for a treatment period of 12 months (Wilcock et al., 2008) and in a Phase 3 study with over 1,600 individuals with mild AD for a treatment period of 18 months (Green et al., 2009). No consistent clinical benefits on cognition were observed in either of these studies. The lack of clinical effects was attributed to the low potency and the absence of effects on CSF Aβ levels, which led to the conclusion that in the absence of central target engagement these studies did not actually test the hypothesis of whether treatment with GSMs might have therapeutic benefits for AD (Wan et al., 2009; Sano, 2010). A derivative of tarenflurbil, CHF5074 (itanapraced, CSP-1103) was also reported to function as a GSM with moderate *in vitro* potency (Aβ42 lowering IC_50_ = 3.6 μM) and some effect on amyloid plaques in Tg2576 mice after a 17-week chronic treatment (Imbimbo et al., 2007). When tested in AD patients in a small 12-week clinical trial with up to 600 mg Q.D. no significant effects on CSF Aβ42 levels and no clinical benefits were recorded (Ross et al., 2013). There were additional preclinical tests and clinical trials performed with this compound which will not be discussed here.

Based on our assessment of publicly disclosed clinical trials for GSMs, nivegacetor is the first brain-penetrant GSM being tested in large Phase 2 clinical trials in individuals with AD. Nivegacetor went through the required entry-into-human enabling toxicological and safety pharmacological studies which were deemed supportive of progressing the molecule into clinical development. Nivegacetor has been examined in a Phase 1 clinical trial in healthy individuals which confirmed central target engagement and supported the further clinical development for the treatment of AD (Lott et al., 2023; Mueggler et al., 2025); the detailed Phase 1 study design and results will be reported elsewhere. Nivegacetor is currently under investigation in two clinical trials: A Phase 2 clinical study evaluating the safety, tolerability, and effects on biomarkers in individuals at risk or at the prodromal stage of AD (NCT06402838) with a treatment duration of 18 months (Tortelli, 2024). In addition, an investigator-initiated study is about to start investigating nivegacetor alone or in combination with Kisunla® (donanemab), an anti-Aβ immunotherapy, in individuals carrying the PSEN1 E280A ADAD mutation (NCT06996730).

Looking at the landscape of AD therapies currently being studied, GSMs target Aβ pathology and their downstream effects by acting complementary to Aβ immunotherapies. While the latter target aggregated forms of Aβ and thereby remove pre-existing Aβ pathology, GSMs act earlier in the Aβ cascade by addressing the *de novo* production of amyloidogenic Aβ species. On this background, it is conceivable that GSMs might find their place in AD therapy as a preventative treatment in individuals with preclinical or early-stage AD, or as follow-on therapy in individuals who have undergone successful Aβ immunotherapy (De Strooper and Karran, 2016; Nordvall et al., 2023).

## 5 Conclusion

Nivegacetor is a novel clinical candidate with potent and selective GSM pharmacology. The compound showed favorable drug-like properties and robust central target engagement in a completed Phase 1 study in healthy individuals. Nivegacetor is the first brain-penetrant GSM being tested in individuals with AD. The results of the ongoing studies will give valuable information on the long-term safety and tolerability of the compound, as well as on its effect on a broad range of biomarkers related to Aβ pathology and tau pathology, neuroinflammation, and neurodegeneration. Furthermore, we will determine whether the clinical data provide insight into the translatability of the preclinical studies performed with the compound.

## Data Availability

The original contributions presented in the study are included in the article/Supplementary Material, further inquiries can be directed to the corresponding author.
