# Supplementary material for: Pharmacology of nivegacetor (RG6289), a potent and selective gamma secretase modulator in clinical development for the treatment of Alzheimer’s disease
Source: Front Pharmacol. 2026 May 28;17:1783414. doi: 10.3389/fphar.2026.1783414 (PMC13254515; doi:10.3389/fphar.2026.1783414)
Supplement: Supplementary file 1 [file Supplementaryfile1.pdf]

Supplementary Material

1. Supplementary Figures and Tables

1.1 Supplementary Figures

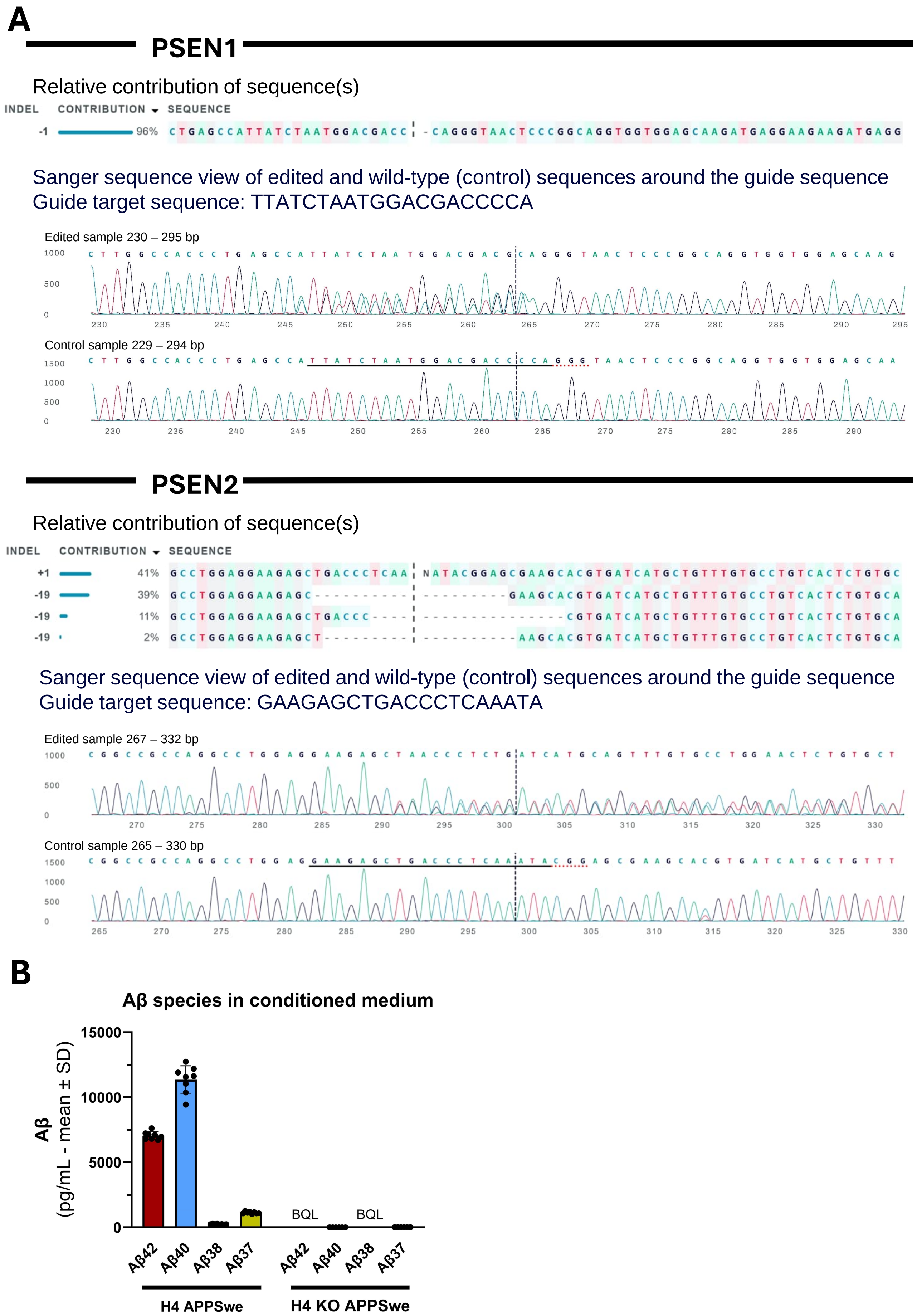

Supplementary Figure 1. H4 KO APPSwe cells properties – gene targeting and Aβ production. Figure legend on next page

Supplementary Figure 1. H4 KO APPSwe cells properties – gene targeting and Aβ production.

H4 APPSwe cells were subjected to targeted disruption of the PSEN1 and PSEN2 genes,respectively, with the Crispr/Cas technology (work carried out by Synthego, Redwood City, USA). Indels of $N \neq n \times 3$ bp were inserted into both copies of the PSEN1 and PSEN2 genes so that expression of functional protein is disrupted. The successful targeting was analyzed by DNA sequencing (carried out by Synthego) and by analysis of conditioned medium for the presence of Aß peptides (carried out at F. Hoffmann-La Roche AG).

(**A**) Sanger sequencing views for both PSEN1 and PSEN2 genes respectively, showing the WT (control) and edited sequences around the guide sequences. The horizontal black underlined region represents the guide sequence. The horizontal red underline is the PAM (protospacer adjacent motif) site. The vertical black dotted line represents the actual cut site. In the edited sequence, inserted or deleted base pairs are indicated with '+'/'-' symbols; insertion/deletion of base pairs which are not multiples of three will result in frameshifts.

(**B**) Analysis of conditioned medium of H4 APPSwe and H4 APPSwe PSEN1/PSEN2 KO cells. H4 APPSwe PSEN1/PSEN2 KO cells show Aß levels that are at/below the detection limit of the AlphaLisa bioassay used, confirming that the targeted disruption of the PSEN1 and PSEN2 genes prevents expression of functional proteins.

BQL: Below quantification limit.

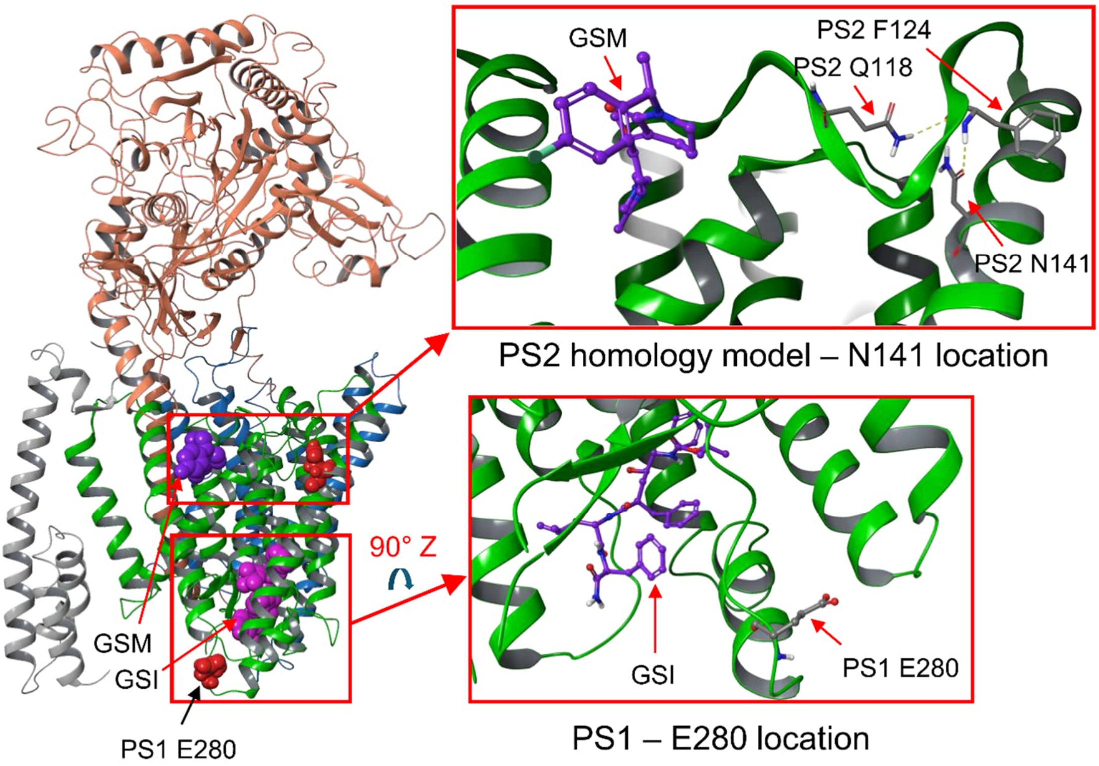

Supplementary Figure 2. Localization of the ADAD mutations PSEN1 E280A and PSEN2 N141I relative to the GSM and GSI binding pockets, based on the cryo-EM structure reported by (Yang et al., 2021).

Structural modeling was based on published Cryo-EM structural data 7D8X (Yang et al., 2021) which shows the gamma secretase structure comprising PS1 with a simultaneously bound gamma secretase inhibitor (GSI) and gamma secretase modulator (GSM). For the PS2 N141I mutation, a homology model of the Cryo-EM structure was built using Schrödinger's Prime software (Schrödinger Release 2024-1: Prime, Schrödinger, LLC, New York, NY, 2025, USA). The relative location of the PSEN1 E280A and PS2 N141I mutations in the gamma secretase structure was visualized using the Maestro software (Schrödinger, New York, USA).

The N135 position corresponds to the PS2 N141 position in the homology model. The full gamma secretase structure is displayed on the left for orientation. The modeling reveals that the PSEN1 E280A mutation is located more distal to the GSM binding pocket compared to the PSEN2 N141I mutation, respectively.

Subunits: NCT = Nicastrin in brown; PEN2 = presenilin enhancer 2 in grey; PS1 = PSEN1 and PS2 = PSEN2 in green; APH1 = Anterior pharynx-1 homolog in cyan (only partially visible).

1.2 Supplementary Tables

Supplementary Table S1: Broad selectivity profile of nivegacetor, radioligand binding and enzymatic assays.

Nivegacetor was profiled on a range of targets at a single concentration of 10 µM. For some targets, nivegacetor was tested multiple times, in those instances the mean result of all tests is reported.

Data is expressed as change of % control, with 0 the lowest and 100 the maximal effect. Numerically negative results are interpreted as lack of activity on the given targets. Radioligand binding experiments employing agonist radiotracers are indicated by an asterisk (*)

**Radioligand Binding Assays** *Receptors: Small Molecule Ligands*				
Target	Source	Non-Specific	Ligand	Mean effect (% Control)
Adenosine receptor 1	Human recombinant (CHO cells)	CPA (10 µM)	[^{3}H]CCPA *	8.5
Adenosine receptor 2a	Human recombinant (HEK293 cells)	NECA (10 µM)	[^{3}H]CGS *	7.1
Adenosine receptor 2b	Human recombinant (HEK293 cells)	NECA (100 µM)	[^{3}H]CPX *	-18.4
Adenosine receptor 3	Human recombinant (HEK293 cells)	IB-MECA (1 µM)	[^{125}I]AB-MECA *	16.4
Adrenergic receptor α1A	Human recombinant (CHO cells)	Epinephrine (100 µM)	[^{3}H]Prazosin	11.8
Adrenergic receptor α1B	Human recombinant (CHO cells)	Phentolamine (10 µM)	[^{3}H]Prazosin	14.9
Adrenergic α2A receptor	Human recombinant (CHO cells)	(-)Epinephrine (100 µM)	[^{3}H]RX821002	5.6
Adrenergic α2B receptor	Human recombinant (CHO cells)	(-)Epinephrine (100 µM)	[^{3}H]RX821002	-12.1
Adrenergic α2C receptor	Human recombinant (CHO cells)	(-)Epinephrine (100 µM)	[^{3}H]RX821002	16.5
Adrenergic β1 receptor	Human recombinant (HEK293 cells)	Alprenolol (50 µM)	[^{3}H](-)CGP 12177 *	-3.7
Adrenergic β2 receptor	Human recombinant (CHO cells)	Alprenolol (50 µM)	[^{3}H](-)CGP 12177	15.0
Adrenergic β3 receptor	Human recombinant (HEK293 cells)	Alprenolol (1 mM)	[^{125}I]Cyanopindolol	4.1
Dopamine D1 receptor	Human recombinant (CHO cells)	SCH 23390 (1 µM)	[^{3}H]SCH23390	4.6
Dopamine D2 receptor (short isoform)	Human recombinant (HEK293 cells)	Butaclamol (10 µM)	[^{3}H]-7-OHDPAT *	17.2
Dopamine D3 receptor	Human recombinant (CHO cells)	(+)Butaclamol (10 µM)	[^{3}H]Methyl-spiperone	0.4
GABA-B receptor (isoform 1b)	Human recombinant (CHO cells)	CGP 52432 (100 µM)	[^{3}H]CGP54626	-10.9
Histamine H1 receptor	Human recombinant (HEK293 cells)	Pyrilamine (1 µM)	[^{3}H]Pyrilamine	14.5
Histamine H2 receptor	Human recombinant (CHO cells)	Tiotidine (100 µM)	[^{125}I]APT	-6.2
Histamine H3 receptor	Human recombinant (CHO cells)	(R)α-Mehistamine (1 µM)	[^{3}H]Nα-oMehistamine *	7.3
Histamine H4 receptor	Human recombinant (HEK293 cells)	Imetit (1 µM)	[^{3}H]Histamine *	-5.9

Supplemental Table S1 (continued): Broad selectivity profile of nivegacetor, radioligand binding and enzymatic assays.

**Radioligand Binding Assays**<br>*Receptors: Small Molecule Ligands*				
Target	Source	Non-Specific	Ligand	Mean effect (% Control)
Melatonin receptor 1	Human recombinant (CHO cells)	Melatonin (1 μM)	[^{125}I]2-Iodomelatonin *	11.1
Melatonin receptor 3	Hamster brain	Melatonin (30 μM)	[^{125}I]2-Iodomelatonin *	20.2
Muscarinic receptor 1	Human recombinant (CHO cells)	Atropine (1 μM)	[^{3}H]Pirenzepine	40.9
Muscarinic receptor 2	Human recombinant (CHO cells)	Atropine (1 μM)	[^{3}H]AFDX384	49.1
Muscarinic receptor 3	Human recombinant (CHO cells)	Atropine (1 μM)	[^{3}H]4-DAMP	12.1
Muscarinic receptor 4	Human recombinant (CHO cells)	Atropine (1 μM)	[^{3}H]4-DAMP	61.0
Opoid receptor δ	Human recombinant (Chem-1 (RBL) cells)	Naltrexone (10 μM)	[^{3}H]DADLE *	11.3
Opoid receptor κ	Rat recombinant (CHO cells)	Naloxone (10 μM)	[^{3}H]U69593 *	29.3
Opoid receptor μ	Human recombinant (HEK293 cells)	Naloxone (10 μM)	[^{3}H]DAMGO *	12.4
Serotonin receptor 5-HT1A	Human recombinant (HEK293 cells)	8-OHDPAT (10 μM)	[^{3}H]8-OHDPAT *	69.3
Serotonin receptor 5-HT1B	Rat cerebral cortex	Serotonin (10 μM)	[^{125}I]CYP (+ 30 μM isoproterenol)	35.4
Serotonin receptor 5-HT1D	Rat recombinant (CHO cells)	Serotonin (10 μM)	[^{3}H]Serotonin *	27.4
Serotonin receptor 5-HT2A	Human recombinant (HEK293 cells)	(±)DOI (1 μM)	[^{125}I](±)DOI *	1.4
Serotonin receptor 5-HT2B	Human recombinant (CHO cells)	(±)DOI (1 μM)	[^{125}I](±)DOI *	40.7
Serotonin receptor 5-HT2C	Human recombinant (HEK293 cells)	(±)DOI (10 μM)	[^{125}I](±)DOI *	-19.5
Serotonin receptor 5-HT3	Human recombinant (CHO cells)	MDL 72222 (10 μM)	[^{3}H]BRL43694	0.5
Serotonin receptor 5-HT4e	Human recombinant (HEK293 cells)	Serotonin (100 μM)	[^{3}H]GR113808	-12.9
Serotonin receptor 5-HT6	Human recombinant (CHO cells)	Serotonin (100 μM)	[^{3}H]LSD *	0.6
Serotonin receptor 5-HT7	Human recombinant (CHO cells)	Serotonin (10 μM)	[^{3}H]LSD *	20.6
Sigma receptor (non-selective)	Human endogenous (Jurkat cells)	Haloperidol (10 μM)	[^{3}H]DTG *	66.7
**Radioligand Binding Assays**<br>*Receptors: Peptides and lipids*				
Angiotensin receptor 1	Human recombinant (HEK293 cells)	Angiotensin-II (10 μM)	[^{125}I][Sar1,Ile8]-AT-II	1.6
Angiotensin receptor 2	Human recombinant (HEK293 cells)	Angiotensin-II (1 μM)	[^{125}I]CGP42112A *	-4.0
Apelin receptor	Human recombinant (CHO cells)	Apelin-13 (1 μM)	[^{125}I] (Glp65,Nle7 5,Tyr77)-apelin-13 *	4.0

Supplemental Table S1 (continued): Broad selectivity profile of nivegacetor, radioligand binding and enzymatic assays.

**Radioligand Binding Assays** *Receptors: Peptides and lipids*				
Target	Source	Non-Specific	Ligand	Mean effect (% Control)
Bombesin-like receptor 3	Human recombinant (CHO cells)	Bn (6-14) (1 µM)	[^{125}I]Bn(6-14) *	21.9
Bradykinin B2 receptor	Human recombinant (CHO cells)	Bradykinin (1 µM)	[^{3}H]Bradykinin *	-2.5
Cannabinoid receptor 1	Human recombinant (CHO cells)	WIN55212-2 (10 µM)	[^{3}H]CP55940 *	-9.5
Cannabinoid receptor 2	Human recombinant (CHO cells)	WIN 55212-2 (5 µM)	[^{3}H]WIN 55212-2 *	9.6
Chemokine receptor type 2	Human recombinant (HEK293 cells)	MCP-1 (10 nM)	[^{125}I]MCP-1 *	5.7
Cholecystokinin receptor 1	Human recombinant (CHO cells)	CCK-8s (1 µM)	[^{125}I]CCK-8s *	-6.5
Cholecystokinin receptor 2	Human recombinant (CHO cells)	CCK-8s (1 µM)	[^{125}I]CCK-8s *	12.0
Corticotropin-releasing factor receptor 1	Human recombinant (CHO cells)	Sauvagine (0.5 µM)	[^{125}I]Sauvagine *	-0.5
Cysteinyl leukotriene receptor 1	Human recombinant (CHO cells)	LTD4 (1 µM)	[^{3}H]LTD4 *	7.0
Endotelin receptor A	Human recombinant (CHO cells)	Endothelin-1 (0.1 µM)	[^{125}I]Endothelin-1 *	9.5
Endotelin receptor B	Human recombinant (CHO cells)	Endothelin-1 (0.1 µM)	[^{125}I]Endothelin-1 *	-12.7
Glucagon receptor	Human recombinant (CHO cells)	Glucagon (1 µM)	[^{125}I]Glucagon *	-9.3
Melanin-concentrating hormone receptor 1	Human recombinant (CHO cells)	Human MCH (0.1 µM)	[^{125}I] [Phe^{13},Tyr^{19}]-MCH *	9.4
Melanocortin 1 receptor	Mouse endogenous (B16-F1 cells)	NDP-α-MSH (1 µM)	[^{125}I]NDP-α-MSH *	-10.6
Melanocortin 3 receptor	Human recombinant (CHO cells)	NDP-α-MSH (1 µM)	[^{125}I]NDP-α-MSH *	-4.8
Melanocortin 4 receptor	Human recombinant (CHO cells)	NDP-α-MSH (1 µM)	[^{125}I]NDP-α-MSH *	0.2
Motilin receptor	Human recombinant (CHO cells)	[Nleu13]-motilin (1 µM)	[^{125}I]Motilin *	-16.3
Neurokinin receptor 1	Human endogenous (U373MG cells)	[Sar^{9},Met(O$_2$)^{11}]-SP (1 µM)	[^{125}I]Substance P LYS3 *	30.7
Neurokinin receptor 2	Human recombinant (CHO cells)	[Nleu^{10}]-NKA (4-10) (0.3 µM)	[125I]NKA *	36.7
Neuropeptide Y receptor 1	Human endogenous (SK-N-MC cells)	NPY (1 µM)	[^{125}I]Peptide YY *	22.6
Nociceptin (orphanin FQ) receptor	Human recombinant (Chem-1 (RBL) cells)	Nociceptin (1 µM)	[^{3}H]Nociceptin *	1.4
Platelet-activating factor receptor	Human recombinant (CHO cells)	C16-PAF (10 µM)	[^{3}H]C$_{18}$-PAF *	59.6
Prostaglandin E2 (EP2) receptor	Human recombinant (HEK293 cells)	PGE$_2$ (10 µM)	[^{3}H]PGE$_2$ *	9.9

Supplemental Table S1 (continued): Broad selectivity profile of nivegacetor, radioligand binding and enzymatic assays.

Target	Source	Non-Specific	Ligand	Mean effect (% Control)
**Radioligand Binding Assays** *Receptors: Peptides and lipids*				
Prostaglandin F2α (FP) receptor	Human recombinant (HEK293 cells)	Cloprostenol (10 μM)	[3H]$PGF_{2α}$ *	10.3
Prostaglandin I2 (IP) receptor	Human recombinant (HEK293 cells)	Iloprost (10 μM)	[3H]Iloprost *	6.5
Somastatin receptor 1	Human recombinant (CHO cells)	Somatostatin-28 (1 μM)	[125I]Tyr_{11}-somatostatin-14 *	-10.9
Somastatin receptor 4	Human recombinant (CHO cells)	Somatostatin-14 (1 μM)	[125I]Tyr_{11}-somatostatin-14 *	21.7
TNF-α receptor	Human endogenous (U-937 cells)	TNF-α (10 nM)	[125I]TNF-α *	4.1
Urotensin receptor (GPR14)	Human recombinant (CHO cells)	Urotensin-II (3 μM)	[125I]Urotensin-II *	44.8
Vasoactive intestinal peptide receptor (VPAC1)	Human recombinant (CHO cells)	VIP (1 μM)	[125I]VIP *	-1.3
Vasopressin receptor V1a	Human recombinant (CHO cells)	AVP (1 μM)	[3H]AVP *	9.0
Vasopressin receptor V2	Human recombinant (CHO cells)	AVP (1 μM)	[3H]AVP *	-3.5
**Radioligand Binding Assays** *Receptors: Nuclear hormone receptors*				
Androgen receptor	Human endogenous (LNCaP cells)	Testosterone (1 μM)	[3H]Methyltrienolone *	-15.7
Estrogen receptor α	Human recombinant (sf9 cells)	Diethylstilbestrol (1 μM)	[3H]Estradiol *	-13.4
Glucocorticoid receptor (GR)	Human endogenous (IM-9 cells)	Triamcinolone (10 μM)	[3H]Dexamethasone *	-10.9
Liver X receptor β (LXRβ)	Human recombinant (*E. coli*)	T0901317 (10 μM)	[3H]T0901317 *	-15.2
Peroxisome proliferator-activated receptor γ (PPAR γ)	Human recombinant (*E. coli*)	Rosiglitazone (10 μM)	[3H]Rosiglitazone *	10.6
Thyroid hormone receptor (non-selective)	Rat liver	Triiodothyronine (1.0 μM)	[125I]Triiodothyronine *	9.1
**Radioligand Binding Assays** *Receptors: Transporters, enzymes*				
5-HT transporter	Human recombinant (CHO cells)	Imipramine (10 μM)	[3H]Imipramine	36.6
Choline transporter (CHT1)	Human recombinant (CHO cells)	Hemicholinium-3 (10 μM)	[3H]Hemicholinium-3	4.6
Dopamine transporter	Human recombinant (CHO cells)	BTCP (10 μM)	[3H]BTCP	38.3
GABA transporter	Rat cerebral cortex	GABA (1 mM)	[3H]GABA (+ 10 μM isoguvacine) (+ 10 μM baclofen)	-17.7
Norepinephrine transporter	Human recombinant (CHO cells)	Desipramine (1 μM)	[3H]Nisoxetine	-1.5
Monoamine oxidase A	Rat cerebral cortex	Clorgyline (1 μM)	[3H]Ro41-1049	9.1

Supplemental Table S1 (continued): Broad selectivity profile of nivegacetor, radioligand binding and enzymatic assays.

**Radioligand Binding Assays** *Ion channels*				
Target	Source	Non-Specific	Ligand	Mean effect (% Control)
AMPA-type glutamate receptor	Rat cerebral cortex	L-glutamate (1 mM)	[^{3}H]AMPA *	2.8
Ca^{2+} channel (L, dihydropyridine site)	Rat cerebral cortex	Nitrendipine (1 µM)	[^{3}H]Nitrendipine	7.4
Ca^{2+} channel (L, diltiazem site) (benzothiazepines)	Rat cerebral cortex	Diltiazem (10 µM)	[^{3}H]Diltiazem	0.9
Ca^{2+} channel (L, verapamil site) (phenylalkylamine)	Rat cerebral cortex	D 600 (10 µM)	[^{3}H]D888	5.3
Ca^{2+} channel (N-type, voltage-gated)	Rat cerebral cortex	ω-conotoxin GVIA (10 nM)	[^{125}I]ω-Conotoxin GVIA	-4.2
GABA-A (non-selective, TBPS)	Rat cerebral cortex	Picrotoxinin (20 µM)	[^{35}S]TBPS	65.7
GABA-A1 receptor (α1,β2,γ2)	Human recombinant (CHO cells)	Muscimol (10 µM)	[^{3}H]Muscimol *	-22.9
Glycine receptor (strychnine-insensitive)	Rat cerebral cortex	Glycine (1 mM)	[^{3}H]MDL 105,519	-5.1
Kainate-type glutamate receptor	Rat cerebral cortex	L-glutamate (1 mM)	[^{3}H]Kainic acid *	-2.8
Na^{+} channel (site 2)	Rat cerebral cortex	Veratridine (300 µM)	[^{3}H]Batrachotoxin	23.7
Nicotinic acetylcholine receptor α4β2	Human recombinant (SH-SY5Y cells)	Nicotine (10 µM)	[^{3}H]Cytisine *	-1.2
Nicotinic acetylcholine receptor (muscle type)	Human endogenous (TE671 cells)	α-Bungarotoxin (5 µM)	[^{125}I]α-Bungarotoxin	10.6
NMDA (PCP site)	Rat cerebral cortex	MK 801 (10 µM)	[^{3}H]TCP	-10.4
NMDA (antagonist radioligand)	Rat cerebral cortex	Lglutamate (100 µM)	[^{3}H]CGP39653	1.3
BZD (central)	Rat cerebral cortex	Diazepam (3 µM)	[^{3}H]Flunitrazepam *	-23.3
SKCa channel (antagonist radioligand)	Rat cerebral cortex	Apamin (100 nM)	[^{125}I]Apamin	-5.1

**Functional Assays**				
Target	Source	Substrate / stimulus	Measured component	Mean effect (% Control)
Abl kinase	Human recombinant (insect cells)	ATP + Ulight-TK peptide (100 nM)	phospho-Ulight-TK peptide	8.7
Acetylcholinesterase	Human recombinant (HEK-293 cells)	Acetylthiocholine (400 µM)	5-thio-2-nitrobenzoic acid	18.6
Angiotensin-converting-enzyme (ACE)	Human recombinant	Mca-Tyr-Val-Ala-Asp-Pro-Ala-Lys-(DNP)-OH (10 µM)	Mca peptides	-11.6
Angiotensin-converting-enzyme 2 (ACE-2)	Human recombinant (murine cells)	Mca-Tyr-Val-Ala-Asp-Pro-Ala-Lys-(DNP)-OH (10 µM)	Mca peptides	3.5
ATPase (Na+/K+)	Porcine cerebral cortex	ATP (2 mM)	Anorganic phosphate (Pi)	1.8

Supplemental Table S1 (continued): Broad selectivity profile of nivegacetor, radioligand binding and enzymatic assays.

Functional Assays				
Target	Source	Substrate / stimulus	Measured component	Mean effect (% Control)
Beta secretase 1 (BACE1)	Human recombinant (mammalian cells)	Mca-S-E-V-N-L-D-A-E-F-R-K(Dnp)-R-R-NH2 (6 μM)	Mca-S-E-V-NL-NH2	3.6
CaMKIIα	Human recombinant	ATP + Ulight-CGSGSGRPRTSSFAEG (50 nM)	phospho-Ulight-CGSGSGRPRTSSFAEG	-4.3
Caspase-3	Human recombinant (*E. coli*)	Benzyloxycarbonyl-Asp-Glu-Val-Asp-AFC (3.6 μM)	AFC	5.4
CDK2 (cycA)	Human recombinant	ATP + Ulight-CFFKNIVTPR TPPPSQGKamide (50 nM)	phospho-Ulight-CFFKNIVTPRTPPPSQGK-amide	2.4
Cyclooxygenase-1	Human recombinant	Arachidonic acid (3μM) + ADHP (25 μM)	Resorufin (oxydized ADHP)	19.7
Cyclooxygenase-2	Human recombinant (Sf9 cells)	Arachidonic acid (1.2 μM) + ADHP (25 μM)	Resorufin (oxydized ADHP)	6.2
Extracellular signal-regulated kinases 2 (ERK2)	Human recombinant (*E. coli*)	ATP + Ulight-CFFKNIVTPR TPPPSQGK-amide (100 nM)	phospho-Ulight-CFFKNIVTPRTPPPSQGK-amide	15.9
FLT-1 kinase (VEGF receptor 1)	Human recombinant (Sf9 cells)	ATP + Ulight-TK peptide (100 nM)	phospho-Ulight-TK peptide	4.3
Fyn kinase	Human recombinant (insect cells)	ATP + biotinyl-βAβAβ AYQAEENTY DEYEN (2 μM)	phosphobiotinyl-βAβAβ AYQAEENTYDEYEN	19.1
Glycogen synthase kinase 3 α (GSK3A)	Human recombinant	ATP + Ulight-CFFKNIVTPR TPPPSQGK-amide (100 nM)	phospho-Ulight-CFFKNIVTPRTPPPSQGK-amide	-16.6
Glycogen synthase kinase 3 β (GSK3B)	Human recombinant	ATP + Ulight-CFFKNIVTPR TPPPSQGK-amide (100 nM)	phospho-Ulight-CFFKNIVTPRTPPPSQGK-amide	1.9
Guanylyl cyclase (activator effect)	Human recombinant	GTP (10 μM) (100 μM SNP for control)	cGMP	-0.6
HIV-1 protease	Protein viral recombinant (*E.coli*)	Antranilyl-HIV (75 μM)	N-terminal tripeptide	5.7
Inducible nitric oxide synthetase	Mouse recombinant (*E. coli*)	L-arginine (100 μM)	NO_2^-	-0.5
Insulin receptor (IRK)	Human recombinant	ATP + Ulight-Poly GAT[EAY(1:1:1)]n (50 nM)	Phosphor-Ulight-Poly GAT[EAY(1:1:1)]n	3.7
Lyn A kinase	Human recombinant (insect cells)	ATP + biotinyl-βAβAβ AKVEKIGEGTYGVVYK (400 nM)	phosphobiotinyl-βAβAβ AKVEKIGEGTYGVVYK	19.1
Matrix metalloprotease 1	Human recombinant (*E. coli*)	DNP-Pro-Cha-Gly-Cys(Me)-His-Ala-Lys(n-Me-Abz)-NH2 (10 μM)	Cys(Me)-His-Ala-Lys(n-Me-Abz)-NH2	5.6
Matrix metalloprotease 2	Human recombinant	NFF-2 (10 μM)	Mca-Arg-Pro-Lys-Pro-Tyr-Ala	-5.4
Matrix metalloprotease 9	Human recombinant	NFF-2 (10 μM)	Mca-Arg-Pro-Lys-Pro-Tyr-Ala	9.0

Supplemental Table S1 (continued): Broad selectivity profile of nivegacetor, radioligand binding and enzymatic assays.

Functional Assays				
Target	Source	Substrate / stimulus	Measured component	Mean effect (% Control)
Matrix metalloprotease 24	Human recombinant (*E. coli*)	Mca-Pro-Leu-Gly-Leu-Dap-Ala-Arg-NH2 (4 μM)	Mca-Pro-Leu-Gly	35.5
Monoamine oxidase A	Human placenta	Kynuramine (0.15 mM)	4-OHquinoline	5.5
Phosphodiesterase 1A (PDE2A1)	Human recombinant (Sf9 cells)	[^{3}H]cAMP + cAMP (2μM)	[^{3}H]5'AMP	5.5
Phosphodiesterase 3B (PDE3B)	Human recombinant (Sf9 cells)	[^{3}H]cAMP + cAMP (0.5μM)	[^{3}H]5'AMP	4.4
Phosphodiesterase 4D2 (PDE4D2)	Human recombinant (Sf9 cells)	[^{3}H]cAMP + cAMP (0.5μM)	[^{3}H]5'AMP	1.9
Phosphodiesterase 5 (PDE5; non-selective)	Human platelets	[^{3}H]cGMP + cGMP (1 μM)	[^{3}H]5'GMP	4.2
Phosphodiesterase 6 (PDE6; non-selective)	Bovine retina	[^{3}H]cGMP + cGMP (2 μM)	[^{3}H]5'GMP	-30.4
p38α kinase	Human recombinant (*E. coli*)	ATP + Ulight-CFFKNIVTPR TPPPSQGK-amide (100 nM)	phospho-Ulight-CFFKNIVTPRTPPPSQGK-amide	-13.3
Zeta-chain-associated protein kinase 70 (ZAP70)	Human recombinant (insect cells)	ATP + biotinyl-βAβAβ ADEEEYFIPP (2 μM)	Phosphobiotinyl-βAβAβ ADEEEYFIPP	4.2
Xanthine oxidase/ superoxide O_2 – scavenging	Purified xanthine oxidase from bovine milk	Hypoxanthine (10 μM)	O_2^- + uric acid	10.5

1.3 Supplementary References

Yang, G., Zhou, R., Guo, X., Yan, C., Lei, J., and Shi, Y. (2021). Structural basis of gamma-secretase inhibition and modulation by small molecule drugs. Cell 184(2), 521-533 e514. doi: 10.1016/j.cell.2020.11.049.
